# Suppressor of Fused regulates the proliferation of postnatal neural stem and precursor cells via a Gli3-dependent mechanism

**DOI:** 10.1242/bio.039248

**Published:** 2019-05-29

**Authors:** Hector G. Gomez, Hirofumi Noguchi, Jesse Garcia Castillo, David Aguilar, Samuel J. Pleasure, Odessa R. Yabut

**Affiliations:** 1 Department of Neurology; 2 Programs in Neuroscience and Developmental Biology, Eli and Edythe Broad Center of Regeneration Medicine and Stem Cell Research, University of California San Francisco, California 94143, USA

**Keywords:** Neurogenesis, Proliferation, Ventricular-subventricular zone, V-SVZ, Sufu, Gli3

## Abstract

The ventricular-subventricular zone (V-SVZ) of the forebrain is the source of neurogenic stem/precursor cells for adaptive and homeostatic needs throughout the life of most mammals. Here, we report that Suppressor of Fused (Sufu) plays a critical role in the establishment of the V-SVZ at early neonatal stages by controlling the proliferation of distinct subpopulations of stem/precursor cells. Conditional deletion of Sufu in radial glial progenitor cells (RGCs) at E13.5 resulted in a dramatic increase in the proliferation of Sox2+ Type B1 cells. In contrast, we found a significant decrease in Gsx2+ and a more dramatic decrease in Tbr2+ transit amplifying cells (TACs) indicating that innate differences between dorsal and ventral forebrain derived Type B1 cells influence Sufu function. However, many precursors accumulated in the dorsal V-SVZ or failed to survive, demonstrating that despite the over-proliferation of Type B1 cells, they are unable to transition into functional differentiated progenies. These defects were accompanied by reduced Gli3 expression and surprisingly, a significant downregulation of Sonic hedgehog (Shh) signaling. Therefore, these findings indicate a potential role of the Sufu-Gli3 regulatory axis in the neonatal dorsal V-SVZ independent of Shh signaling in the establishment and survival of functional stem/precursor cells in the postnatal dorsal V-SVZ.

## Abstract (precis)

**Summary:** Conditional deletion of Sufu causes dramatic expansion of neural stem/precursor cells in the neonatal ventricular-subventricular (SVZ) zone. This defect occurs through a Gli3-dependent mechanism resulting in the downregulation of Shh signaling.

## INTRODUCTION

Tissue-specific stem cell niches persist at postnatal stages as the source of multiple cell types throughout the life of most animal species. In the mammalian forebrain, particularly in rodents, the ventricular-subventricular zone (V-SVZ) lining the lateral ventricles is a prominent postnatal stem cell niche capable of generating neuronal and glial cell progenies. The V-SVZ are the source of inhibitory neurons (interneurons) that migrate and integrate within the neural network of the olfactory bulb (OB) to influence behaviors including predator avoidance and sex-specific responses (Sakamoto et al., 2011). The adult V-SVZ is composed of Type B1 cells, transit amplifying Type C cells (TACs), Type A cells and a monolayer of ependymal cells along the ventricular wall. Type B1 cells are the primary neural stem cells (NSC) of the V-SVZ, capable of generating TACs that divide into immature cell types that migrate into various forebrain structures where they mature (Lim and Alvarez-Buylla, 2016). Specifically, neurogenic TACs differentiate into immature neurons or Type A cells that migrate through the rostral migratory stream (RMS) and differentiate into molecularly distinct interneuron subtypes of the OB circuitry.

Radial glial cells (RGC) in the lateral ganglionic eminence (LGE) and the neocortex of the embryonic ventral and dorsal forebrain, respectively, produce Type B1 cells (Young et al., 2007). The progeny of RGCs originating from these two regions are identifiable in the V-SVZ. Type B1 cells derived from neocortical RGCs typically produce TACs expressing the transcription factor T-Box Brain Protein 2 (Tbr2) (Brill et al., 2009). On the other hand, Type B1 cells derived from LGE RGCs produce TACs expressing the transcription factor Genetic Screen Homeobox 2 (Gsx2) (Stenman et al., 2003) were significantly increased. However, regulatory mechanisms involved in establishing the production of these Type B1 cell lineages at early neonatal stages are largely unclear. Ensuring the proper timing of differentiation and the number of TACs produced at this early stage are critical to avoid early exhaustion of stem cell sources necessary for later adaptation and homeostatic needs, but also to avoid malformations and tumorigenesis. Therefore, further elucidation of the mechanisms controlling the production of specific cell lineages in the neonatal V-SVZ have far-reaching implications.

Suppressor of Fused (Sufu) is a cytoplasmic protein known for antagonizing Sonic hedgehog (Shh) signaling activity (Ramsbottom and Pownall, 2016). In the developing mammalian forebrain, Sufu modulates Shh signaling to control the proliferation, specification and differentiation of RGCs and their progenies (Yabut et al., 2015, 2016; Yabut and Pleasure, 2018). Sufu exerts this role by controlling the activity of Gli transcription factors, the Shh signaling effectors, by proteolytic processing or protein stabilization to promote the repressor function of Gli and the eventual downregulation of Shh signaling target gene expression. Given the diverse roles of Sufu during corticogenesis in regulating RGCs, which eventually generate or transform to Type B1 cells in the postnatal brain, we wondered whether Sufu function in RGCs influence the generation of Type B1 cells and their progenies, and the establishment of the V-SVZ at neonatal stages. To investigate this, we employed a Cre-loxP approach to selectively delete Sufu in forebrain RGCs that give rise to Type B1 cells in the V-SVZ of the mouse forebrain. We found that loss of Sufu caused a dramatic cellular expansion in the dorsal V-SVZ by postnatal day (P) 7 of the neonatal mouse and persists through early adult stages. This resulted in excess production of Sox2+ Type B1 cells at the expense of TACs. We found that these defects were partially due to a decrease in Gli3 expression, but not due to Shh signaling activation. Taken together, these studies indicate that Sufu plays a critical role in regulating neural cell precursor generation in the neonatal forebrain.

## RESULTS

### Expansion of the V-SVZ in the P7 *hGFAP-Cre;Sufu^fl/fl^* mice

**Fig. 1.**
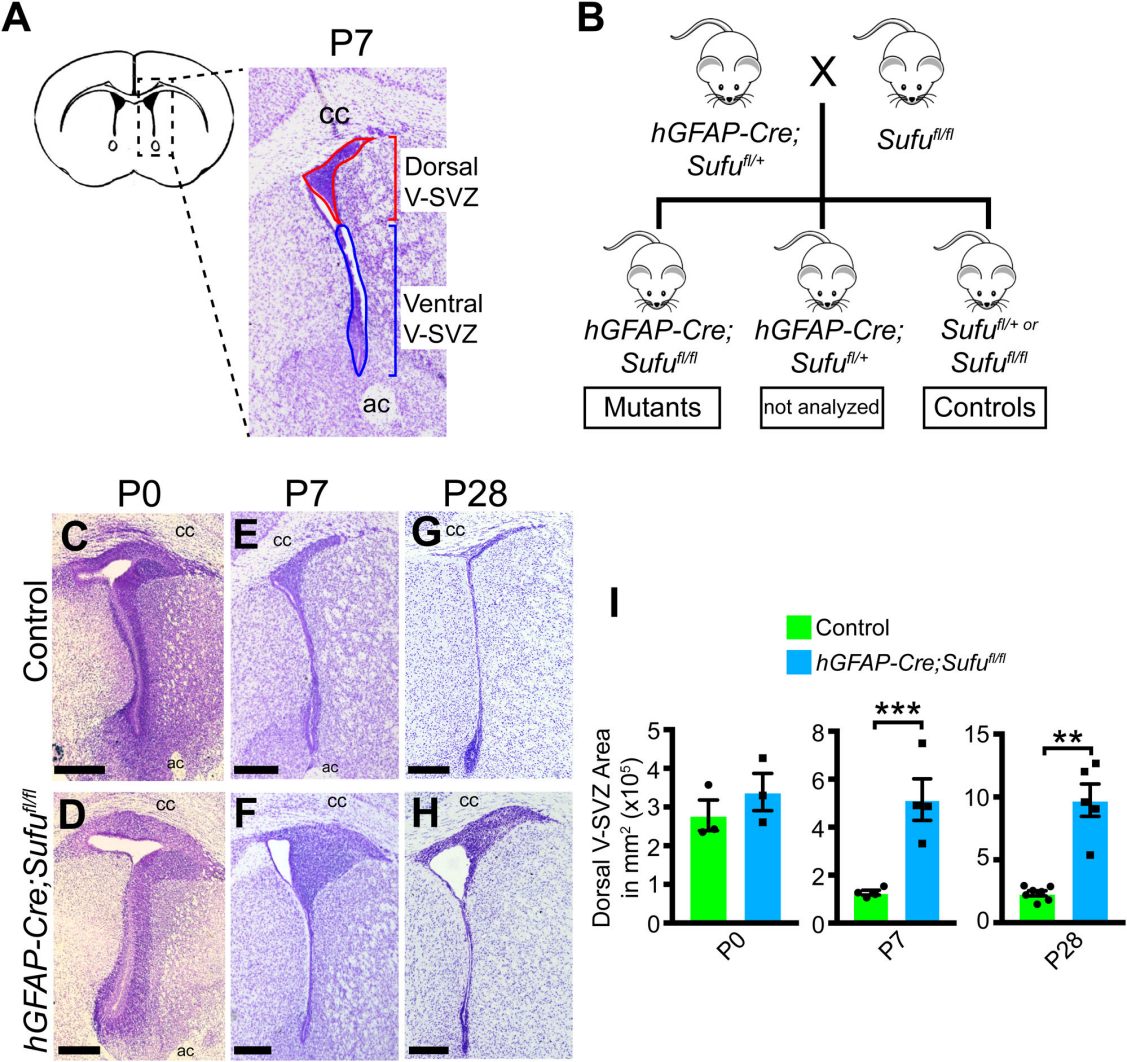
**Loss of Sufu causes an expansion of dorsal V-SVZ cells at early postnatal and adult stages.** (A) Schematic diagram of the P7 dorsal and ventral V-SVZ areas analyzed in these studies. (B) Illustration of the breeding scheme used to generate conditional Sufu knockouts and controls for analysis. (C–H) Cresyl-Violet staining of coronal sections of the P0, P7 and P28 *hGFAP-Cre;Sufu^fl/fl^* and control littermates. No anatomical or structural difference in V-SVZ between the two genotypes was observed at P0, whereas the dorsal V-SVZ is obviously enlarged in the P7 and P28 mutant mice unlike controls. (I) Quantification of V-SVZ area shows no significant difference between the size of the V-SVZ of P0 *hGFAP-Cre;Sufu^fl/fl^* mice and controls (*n*=3 controls/mutants). Quantification of mutant dorsal V-SVZ area of P7 (*n*=4 controls/mutants) and P28 (*n*=7 controls; *n*=5 mutants) shows a statistically significant increase compared to controls. ***P-value≤0.01; **P-value≤0.03. V-SVZ, ventricular-subventricular zone; ac, anterior commissure; cc, corpus callosum. Scale bars: 250 µm.

The postnatal V-SVZ structure is composed of distinct dorsal and ventral domains (Fig. 1A). The rudimentary dorsal and ventral domains can be distinguished anatomically and molecularly at birth. The wild-type dorsal V-SVZ domain expresses dorsal V-SVZ marker, Pax6, while the lateral wall along the ventral V-SVZ domain expresses the marker, Dlx2 (Fig. S1; Brill et al., 2008). These areas are densely populated and, in the case of the ventral V-SVZ, are composed of several cell layers (Fig. 1C). Over time, a progressive reduction in V-SVZ cell density occurs (Fig. 1E,G). The ventral V-SVZ forms a one-cell-layer-thick structure, while the area occupied by the dorsal V-SVZ dramatically decreases (Fig. 1I). These observations indicate that critical regulatory events are actively shaping the V-SVZ cellular structure at early neonatal stages.

Type B1 cells are produced in late embryonic stages from RGCs of the embryonic forebrain and generate the majority of cells populating the V-SVZ. We previously showed that embryonic regulation of RGC proliferation and specification are tightly controlled by the Shh signaling pathway at embryonic stages. To determine how Shh signaling affects Type B1 cells in the developing V-SVZ, we generated a conditional mouse knockout for Sufu, a known antagonist of Shh signaling using the hGFAP-Cre mouse line in which Cre recombinase is specifically active in RG cells of the late stage embryonic forebrain, at E13.5 (Zhuo et al., 2001). Generating the *hGFAP-Cre;Sufu^fl/fl^* mice (Fig. 1B) allowed us to target Sufu deletion in RGCs from all progenitor domains of the dorsal and ventral forebrains. At P0, we examined coronal sections from V-SVZ regions of *hGFAP-Cre;Sufu^fl/fl^* and control littermates and found no obvious anatomical differences (Fig. 1C,D) and that the dorsal and ventral V-SVZ domains correctly formed in the mutant V-SVZ, as determined by the clear demarcation of Pax6+ dorsal V-SVZ and Dlx2+ ventral V-SVZ domains (Fig. S1). By P7, we found a dramatic enlargement of the dorsal V-SVZ in mutant mice compared to control littermates, while the ventral V-SVZ was comparable between the two genotypes (Fig. 1E,F; data not shown). Quantification of the overall dorsal V-SVZ area confirmed that no significant difference in the overall size of the dorsal V-SVZ was observed between controls and mutants at P0 (Fig. 1I; 278,512±39,546 µm^2^ for *n*=3 controls and 338,946±48,133 µm^2^ for *n*=3 mutants; *P*-value=0.369). However, the mutant dorsal V-SVZ was expanded approximately three-fold compared to control littermates by P7 (Fig. 1I; 126,984±9915 µm^2^ for *n*=4 controls and 514,863±86,674 µm^2^ for *n*=4 mutants; *P*-value =0.0043). By P28, despite a considerable reduction in the overall area of the dorsal V-SVZ in both genotypes, the mutant dorsal V-SVZ remained significantly enlarged compared to controls (Fig. 1G-I; 23,323±2067 µm^2^ for *n*=7 controls and 97,439±12,890 µm^2^ for *n*=5 mutants; *P*-value=0.0001). These observations indicate that Sufu does not play a role in the initial patterning of the dorsal and ventral V-SVZ domains. However, Sufu appears to control the expansion of dorsal V-SVZ cell types at neonatal stages likely influencing the final number of cells residing in this domain at early adult stages.

### Accumulation of proliferating cells in the dorsal V-SVZ of the P7 *hGFAP-Cre;Sufu^fl/fl^* mice

**Fig. 2.**
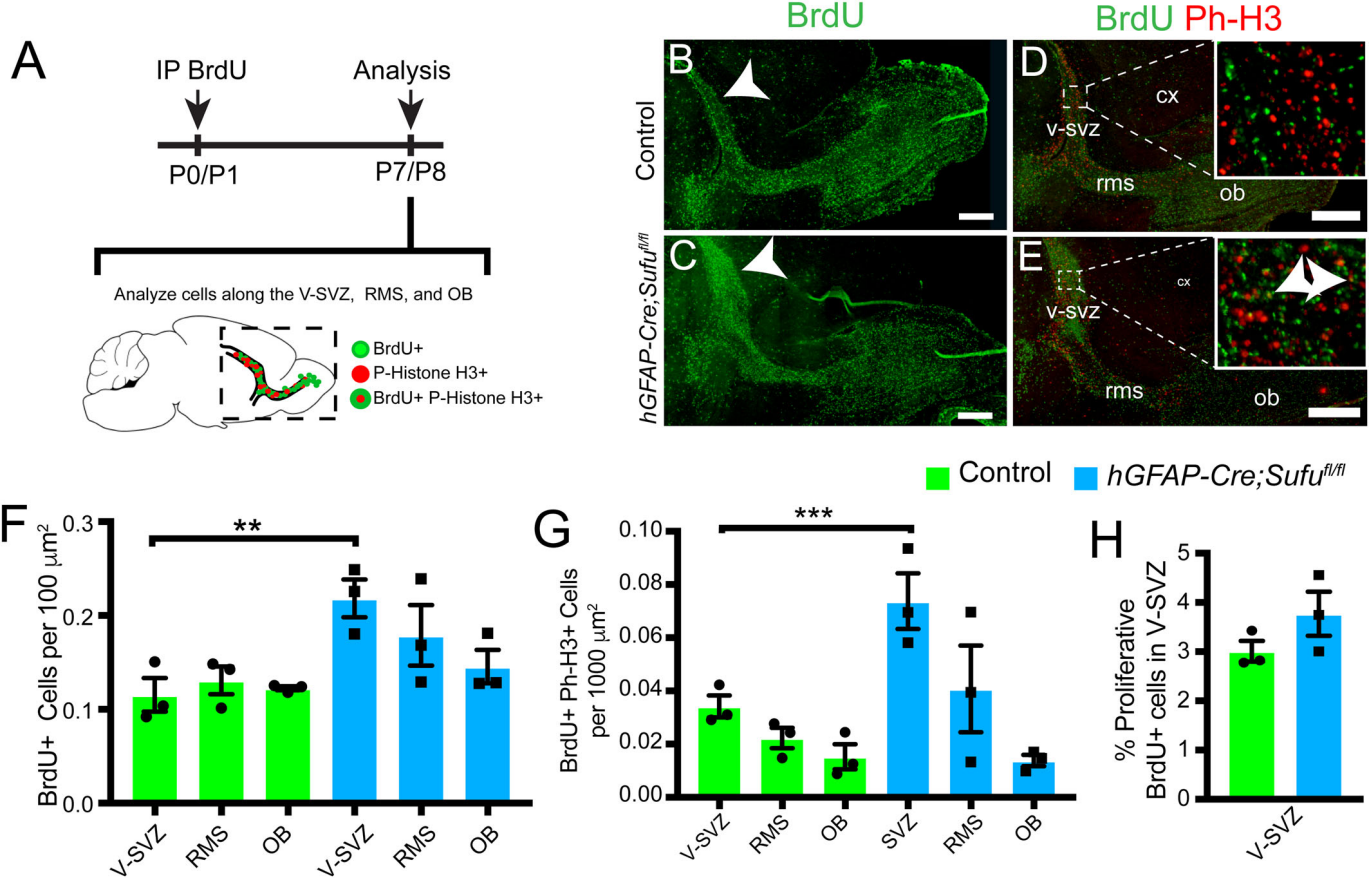
**Cells generated at neonatal stages accumulate in the dorsal V-SVZ of the P7 *hGFAP-Cre;Sufu^fl/fl^* mice and remained proliferative.** (A) Schematic of BrdU-labeling experiments to identify proliferating cells. Intraperitoneal injections (IP) of S-phase label, BrdU, were administered to P0 or P1 littermates and quantification of double-labeled BrdU+ and Phospho-Histone H3 (Ph-H3+) cells in the V-SVZ, RMS, and OB of sagittal sections was performed 7 days later (either P7 or P8). (B,C) Immunofluorescence staining with anti-BrdU shows successful migration of actively proliferating progenitors from the V-SVZ through the RMS, and into the OB of P7 *hGFAP-Cre;Sufu^fl/fl^* mice and control littermates. Arrowheads indicate an observable increase in BrdU+ cells in the P7 *hGFAP-Cre;Sufu^fl/fl^* dorsal V-SVZ compared to controls. (D,E) Immunofluorescence staining with anti-Ph-H3, a mitotic marker, and anti-BrdU shows a visible increase in BrdU+ and Ph-H3+ double-labeled cells (arrowheads) in the dorsal V-SVZ of P7 *hGFAP-Cre;Sufu^fl/fl^* mice compared to controls. (F) Quantification confirms a significant increase in BrdU+ cells in the P7 *hGFAP-Cre;Sufu^fl/fl^* dorsal V-SVZ compared to controls whereas no significant differences in the number of BrdU+ cells were observed in the RMS or OB (*n*=3 controls/mutants). (G) Quantification of BrdU+ and Ph-H3+ double-labeled cells verified an increase in the dorsal V-SVZ of the P7 *hGFAP-Cre;Sufu^fl/fl^* mice compared to controls; however, no significant increase was quantified in the RMS or OB of P7 *hGFAP-Cre;Sufu^fl/fl^* mice compared to controls. (H) Percentage calculation of BrdU+ cells that remained proliferative by Ph-H3 in the V-SVZ show no significant difference between P7 *hGFAP-Cre;Sufu^fl/fl^* mice compared to controls (*n*=3 controls/mutants). **P-value≤0.03; ***P-value≤0.01. V-SVZ, ventricular-subventricular zone; RMS, rostral migratory stream; OB, olfactory bulb; CX, cortex. Scale bars: 500 µm.

The dorsal V-SVZ is populated by actively proliferating precursors, including immature Type A cells that divide and migrate into the OB. To examine whether the increase in cell number in the P7 *hGFAP-Cre;Sufu^fl/fl^* dorsal V-SVZ is due to the failed migration of Type A cells, we labeled proliferating precursors in the V-SVZ of either P0 or P1 littermates by intraperitoneal injection of 5-bromo-2-deoxyuridine (BrdU) and examined the location of BrdU-labeled (BrdU+) cells 7 days later (P7 or P8) (Fig. 2A). Proliferating cells in the dorsal V-SVZ cells were labeled with BrdU at P1 and include TACs destined to differentiate into Type A cells that will migrate anteriorly through the RMS and finally to the OB. Thus, we were able to trace the location of BrdU+ cells along this migratory route over time in sagittal sections of P7 brains (Fig. 2A). As expected, BrdU+ cells were observed in the V-SVZ, the RMS and the OB of control mice, indicating that V-SVZ cells at P1 successfully migrated into the OB by P7 (Fig. 2B). Similarly, we found BrdU+ cells in the V-SVZ, RMS, and OB of P7 *hGFAP-Cre;Sufu^fl/fl^* brains (Fig. 2C). However, an obvious increase in BrdU+ cells were observed in the P7 *hGFAP-Cre;Sufu^fl/fl^* dorsal V-SVZ (arrowhead, Fig. 2C) but not in controls (arrowhead, Fig. 2B). Quantification of BrdU+ cells resulted in a significant increase in the P7 *hGFAP-Cre;Sufu^fl/fl^* V-SVZ compared to controls (Fig. 2F; 0.1154±0.01794 cells per 100 µm^2^ for *n*=3 controls and 0.2183±0.02015 cells per 100 µm^2^ for *n*=3 mutants; *P*-value=0.0189), whereas no significant difference between controls and mutants were quantified in the RMS (0.1308±0.01477 cells per 100 µm^2^ for *n*=3 controls and 0.1789±0.03221 cells per 100 µm^2^ for *n*=3 mutants; *P*-value=0.2463) and OB (0.1225±0.002195 cells per 100 µm^2^ for *n*=3 controls and 0.1457±0.01775 cells per 100 µm^2^ for *n*=3 mutants; *P*-value=0.2650). Overall, these observations indicate that cells within the dorsal V-SVZ of mutant mice were able to migrate despite of the accumulation of BrdU+ cells in the mutant dorsal V-SVZ.

### Persistent cell proliferation in the dorsal V-SVZ of the P7 *hGFAP-Cre;Sufu^fl/fl^* mice

NSCs in the V-SVZ include slowly-dividing quiescent populations able to retain S-phase labels such as BrdU – and are referred to as label-retaining cells – for extended periods (Cotsarelis et al., 1990; Codega et al., 2014). To exclude the possibility that BrdU+ cells in the dorsal V-SVZ are label-retaining quiescent NSCs (qNSC), we examined the proportion of cells that remained proliferative by immunostaining with the mitotic marker Phospho-Histone H3 (Ph-H3) following BrdU labeling (Fig. 2A). We found that, unlike controls (Fig. 2D), many of the accumulated BrdU+ cells in the P7 *hGFAP-Cre;Sufu^fl/fl^* dorsal V-SVZ expressed Ph-H3, and were therefore still proliferative (Fig. 2E). Quantification of double-labeled cells verified the presence of a significantly higher proportion of Ph-H3+ and BrdU+ double-labeled cells in the P7 *hGFAP-Cre;Sufu^fl/fl^* dorsal V-SVZ (Fig. 2G, 0.003417±0.0004118 cells per 100 µm^2^ for *n*=3 controls and 0.007374±0.001042 cells per 100 µm^2^ for *n*=3 mutants; *P*-value=0.0242). However, the proportion of proliferating BrdU+ cells did not significantly differ between controls and mutants (Fig. 2H; 3.008±0.2103% of BrdU+ cells in for *n*=3 controls and 3.771±0.4467% BrdU+ cells for *n*=3 mutants; *P*-value=0.1975). Our findings indicated that loss of Sufu resulted in the continuous proliferation of cells within the dorsal V-SVZ of the P7 *hGFAP-Cre;Sufu^fl/fl^* mice.

### Loss of Sufu drives the proliferation of Type B1 cells in the dorsal V-SVZ

**Fig. 3.**
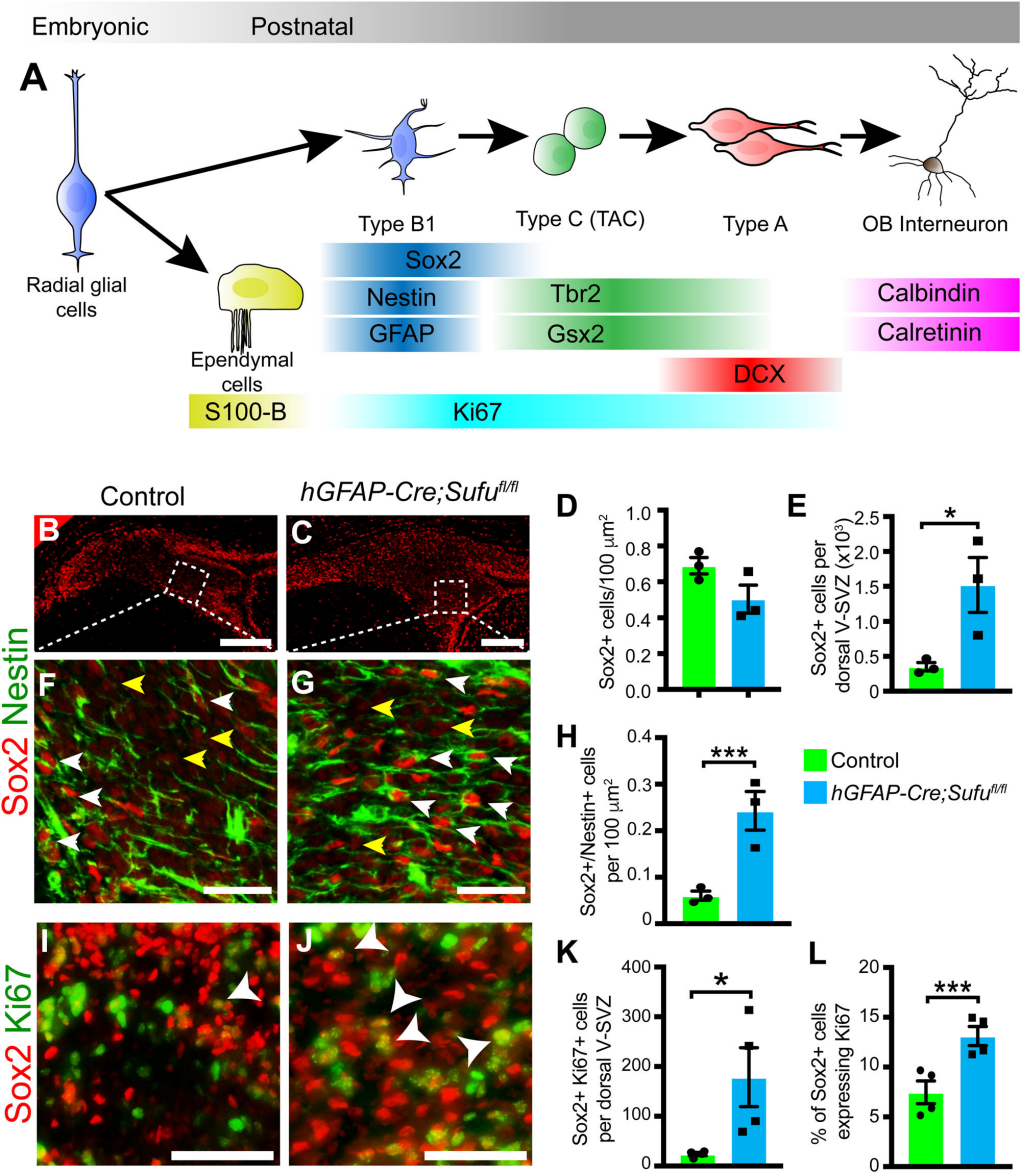
**Loss of Sufu drives the proliferation of multipotent Type B cells in the dorsal V-SVZ.** (A) Schematic showing the lineage of V-SVZ cell types and the markers that are enriched at each stage of cell development. Radial glial cells of the embryonic forebrain give rise to ependymal cells and Type B1 cells. Type B1 cells give rise to rapidly dividing transit amplifying cells (TAC; Type C) which differentiate into Type A cells that migrate into the OB to mature into functional interneurons. (B,C) Immunofluorescence staining with Type B cell marker, Sox2, shows an increase in cells with high expression of Sox2 (Sox2+^high^) in the dorsal V-SVZ of P7 *hGFAP-Cre;Sufu^fl/fl^* mice (C) compared to controls (B). Scale bars: 250 µm. Boxed inset pertains to high magnification images in F and G. (D,E) Quantification of Sox2+^high^ cells in the dorsal V-SVZ showed no significant differences per 100 µm^2^ (D), but showed a significant increase in the number of Sox2+^high^ cells per dorsal V-SVZ (E) in the P7 *hGFAP-Cre;Sufu^fl/fl^* mice, indicating that Sox2+^high^ cells increased in proportion to the expanding dorsal V-SVZ of mutant mice. (*n*=3 controls/mutants). (F,G) Double-immunofluorescence staining with Sox2 and Nestin shows that Sox2+^high^ cells (white arrowheads) in the control (E) and mutant dorsal V-SVZ (F) also co-express the neural stem cell marker, Nestin, and likely represent multipotent Type B cells. Cells expressing low levels of Sox2 (Sox2+^low^) are typically away from the periphery of the control dorsal V-SVZ and do not express Nestin (yellow arrowheads). Notably, there is an increase in Nestin+ Sox2+^high^ double-labeled cells in the mutant dorsal V-SVZ beyond the periphery of the mutant dorsal V-SVZ (F) (*n*=3 controls/mutants). Scale bars: 50 µm. (H) Quantification of Nestin+ Sox2+^high^ double-labeled cells shows a significant increase in the P7 *hGFAP-Cre;Sufu^fl/fl^* dorsal V-SVZ (*n*=3 controls/mutants). (I,J) Immunofluorescence staining against Sox2 and the proliferation marker, Ki67 shows a visible increase in the number of double-labeled cells of the P7 *hGFAP-Cre;Sufu^fl/fl^* dorsal V-SVZ (I) compared to controls (H). Arrowheads mark double-labeled cells. Scale bars: 50 µm. (K,L) Quantification of double-labeled Ki67+ and Sox2+ cells verify a significant increase in the density (K) and percentage (L) of proliferating Sox2+^high^ cells in the P7 *hGFAP-Cre;Sufu^fl/fl^* dorsal V-SVZ compared to controls (*n*=4 controls/mutants). *P-value ≤0.05; ***P-value ≤0.01.

Given the abnormal cell expansion and the continuous proliferation of cells in the dorsal V-SVZ of the P7 *hGFAP-Cre;Sufu^fl/fl^* mice, we analyzed the behavior of specific cell types through labeling with V-SVZ cell specific markers (Fig. 3A). First, we conducted immunostaining with Sox2, a transcription factor highly expressed in the dorsal V-SVZ Type B1 cells (Ellis et al., 2004). At P7, Sox2-expressing (Sox2+) cells were present and accumulated along the lining of the ventricular wall and the periphery of the dorsal V-SVZ of both control and mutant mice (Fig. 3B,C). High magnification analysis of the dorsal V-SVZ showed two distinct populations of Sox2+ cells (Fig. 3F,G): cells expressing high levels of Sox2 (Sox2+^high^, white arrowheads) and cells expressing low levels of Sox2 (Sox2+^low^, yellow arrowheads). Sox2+^high^ cells co-expressed Nestin (Nestin+), indicating that Sox2+^high^ cells are proliferating Type B1 Cells (Codega et al., 2014). Thus we focused our subsequent analysis on Sox2+^high^ cells. These cells were increased in the P7 *hGFAP-Cre;Sufu^fl/fl^* dorsal V-SVZ, particularly in regions where Sox2+^high^ cells were typically scant in the controls (boxed areas, Fig. 3B,C,F,G). When quantified, we did not find any significant difference per unit area in the total number of Sox2+^high^ cells (Fig. 3D, 0.6895±0.04573 cells per 100 µm^2^ for *n*=3 controls and 0.5037±0.0773 cells per 100 µm^2^ for *n*=3 mutants; *P*-value=0.1074). However, we found that the total number of Sox2+^high^ cells dramatically increased per dorsal V-SVZ of mutant mice (Fig. 3E, 351.8±59.82 cells, *n*=3 controls and 1521±391.5 cells, *n*=3 mutants; *P*-value=0.0418). Further quantification of Sox2+^high^ Nestin+ cells showed that Type B1 cells specifically increased in the dorsal V-SVZ of mutant mice (Fig. 3H; 0.06028±0.009856 cells per 100 µm^2^ in controls and 0.2426±0.04155 cells per 100 µm^2^ in mutants, *n*=3 control/mutant mice, *P*-value=0.0130). These findings showed that Sox2+^high^ Type B1 cells proliferated proportionately as the dorsal V-SVZ expanded. Examination of proliferating cells marked by the expression of Ki67 (Ki67+) showed a visibly high number of Sox2+^high^ cells co-expressing Ki67 in the dorsal V-SVZ of P7 *hGFAP-Cre;Sufu^fl/fl^* mice unlike controls (Fig. 3I,J). Our quantification confirmed this observation, in which we found a dramatic increase in double-labeled Sox2+^high^ and Ki67+ cells in the dorsal V-SVZ of P7 *hGFAP-Cre;Sufu^fl/fl^* dorsal V-SVZ, (Fig. 3K; 24.13±3.21 cells, *n*=4 controls; 178.6±59.11 cells, *n*=4 mutants; *P*-value=0.0401). Furthermore, a significant percentage of Sox2+^high^ cells were Ki67+ (Fig. 3L; 7.472±1.141% cells *n*=4 controls; 13.12±0.9569%, *n*=4 mutants; *P*-value=0.0091). Thus, loss of Sufu promoted Type B1 cell proliferation, which likely contributed to the significant expansion of the dorsal V-SVZ in *hGFAP-Cre;Sufu^fl/fl^* mice.

### Reduced transit amplifying cells in the dorsal V-SVZ of P7 *hGFAP-Cre;Sufu^fl/fl^* mice

**Fig. 4.**
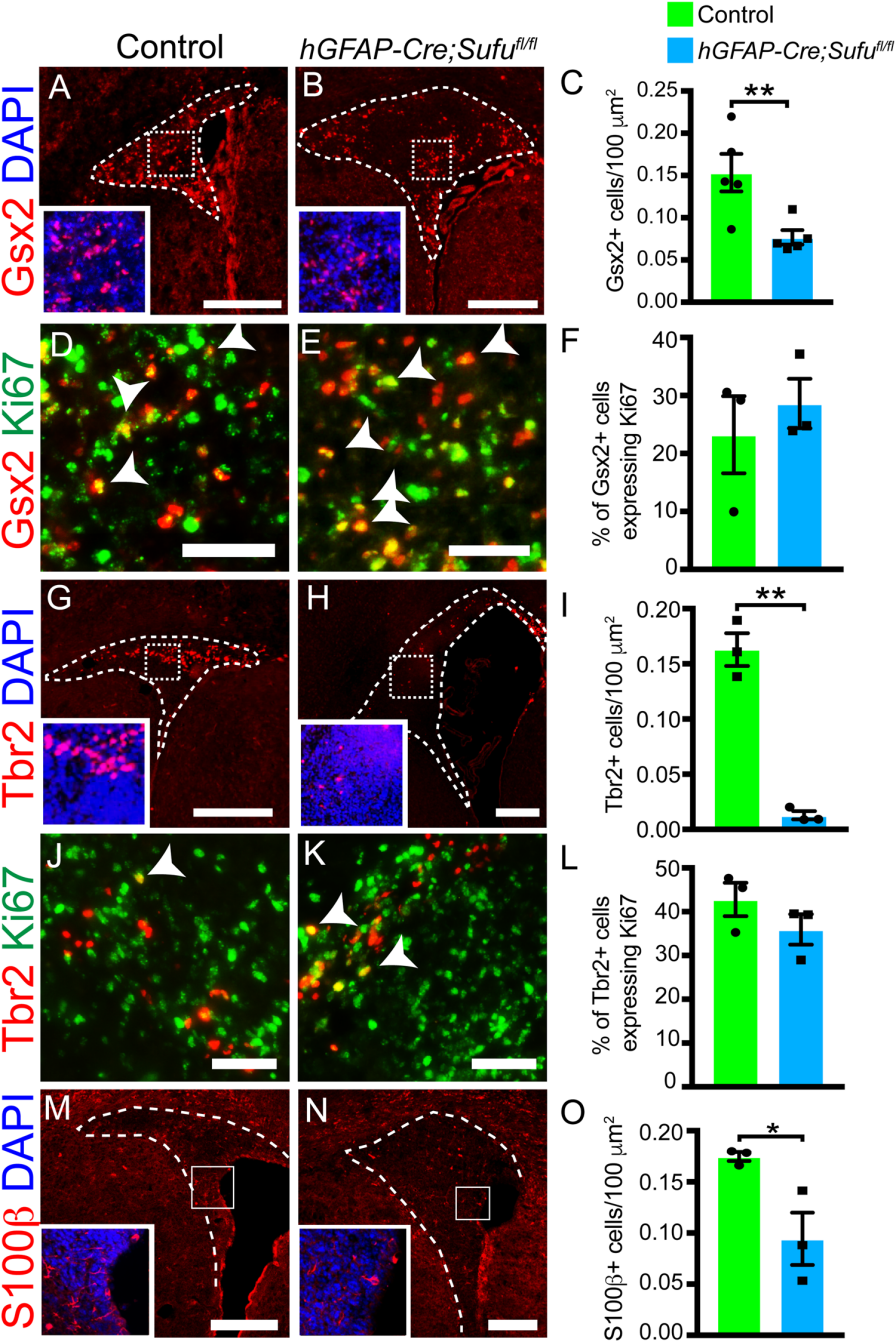
**Sufu deletion causes a reduction in transit amplifying cells (TACs) and ependymal cells in the P7 *hGFAP-Cre;Sufu^fl/fl^* dorsal V-SVZ.** (A,B) Immunofluorescence staining against Gsx2, a marker for TACs, shows that Gsx2+ cells are present in both control (A) and mutant (B) V-SVZ. Scale bars: 250 µm. (C) Quantification of Gsx2+ cells in the P7 dorsal V-SVZ shows a significant reduction in the density of Gsx2+ cells in the mutant mice compared to controls (*n*=5 controls/mutants). (D,E) Double immunofluorescence staining against Gsx2, co-labeled with the proliferation marker Ki67, shows that proliferating Gsx2+ cells are present in both P7 control and *hGFAP-Cre;Sufu^fl/fl^* dorsal V-SVZ. Arrowheads mark double-labeled cells. Scale bars: 50 µm. (F) Quantification of proliferating Gsx2+ cells, verify no significant differences between control and mutant dorsal V-SVZ (*n*=3 controls/mutants). (G,H) Immunofluorescence staining against dorsal forebrain neurogenic progenitor cell marker, Tbr2, shows a visible decrease in the number of Tbr2+ cells in the P7 *hGFAP-Cre;Sufu^fl/fl^* dorsal V-SVZ (H) compared to controls (G). Scale bars: 250 µm. (I) Quantification of Tbr2+ cells in the dorsal V-SVZ shows a significant decrease in the density of Tbr2+ precursors in the P7 *hGFAP-Cre;Sufu^fl/fl^* mice compared to controls (*n*=3 controls/mutants). (J,K) Double immunofluorescence staining against Ki67 and Tbr2, shows proliferating Tbr2+ cells in both control (J) and mutant (K) P7 dorsal V-SVZ. Scale bars: 50 µm. Arrowheads mark double-labeled cells. (L) Quantification of proliferating Tbr2+ and Ki67+ cells, verify no significant differences between control and mutant dorsal V-SVZ (*n*=3 controls/mutants). (M,N) Immunofluorescence staining against ependymal cell marker, S100β, shows a decrease in the density of S100β+ cells in the P7 *hGFAP-Cre;Sufu^fl/fl^* dorsal V-SVZ compared to controls. Scale bars: 200 µm. (O) Quantification of S100β+ cells in the dorsal V-SVZ shows a significant decrease in the density of ependymal cells in the mutant P7 *hGFAP-Cre;Sufu^fl/fl^* mice compared to controls (*n*=3 controls/mutants). *P-value ≤0.05; **P-value ≤0.03.

Type B1 cells in the dorsal V-SVZ generate neurogenic TACs (Fig. 3A). Thus, we investigated how the increase in proliferating Sox2+^high^ cells affected TACs in the dorsal V-SVZ by immunostaining with TAC-specific markers, Gsx2 or Tbr2 (Brill et al., 2009; López-Juárez et al., 2013). At P7, we observed weak Gsx2 expression in GFAP+ cells in the ventricular lining (Fig. S2A–F, blue arrows) whereas strong Gsx2 expression was detected in GFAP- cells in the ventricular lining and outside this region (Fig. S2A–F, white arrows) in both P7 control and *hGFAP-Cre;Sufu^fl/fl^* dorsal V-SVZ. These indicate that Gsx2+/GFAP+ cells in the ventricular lining represented Type B1 cells that have acquired a neuronal precursor fate. The increase in Gsx2 expression, coupled with the loss in GFAP expression, likely marks further differentiation of Type B1 cells into TACs as they migrate away from the ventricular lining. Interestingly, we observed fewer Gsx2+ cells in the ventricular lining of the *hGFAP-Cre;Sufu^fl/fl^* dorsal V-SVZ, pointing to a reduction in the formation of Gsx2+ TACs. Indeed, we found obvious differences in the overall distribution of Gsx2+ cells between the dorsal V-SVZ of control and *hGFAP-Cre;Sufu^fl/fl^* mice. Visibly, Gsx2+ cells were sparsely distributed in the mutant dorsal V-SVZ unlike controls (Fig. 4A,B). Quantification confirmed a significant reduction in the density of Gsx2+ cells in mutants compared to controls (Fig. 4C, 0.1533±0.02214 cells per 100 µm^2^ for *n*=5 controls, 0.07684±0.008522 cells per 100 µm^2^ for *n*=5 mutants; *P*-value=0.0122), although the overall number of Gsx2+ cells in the dorsal V-SVZ did not significantly differ (Fig. S2M, 98.6±14.38 cells per dorsal V-SVZ in *n*=5 controls, 146.7±21.38 cells per dorsal V-SVZ in *n*=5 mutants; *P*-value=0.098). Also, the proliferative capacity of Gsx2+ TACs, as determined by co-labeling with Ki67, did not significantly differ between mutant and control dorsal V-SVZ (Fig. 4D,F; 23.24±6.655% of cells in controls, 28.62±4.27% of cells in mutants; *n*=3 control/mutant mice; *P*-value=0.5337). This indicates that loss of Sufu did not disrupt the ability of Gsx2+ cells to proliferate.

We also analyzed Tbr2-expressing cells, none of which co-localized with GFAP (Fig. S2G–L, white arrows), indicating that Tbr2+ cells at neonatal stages have fully acquired the fate of TACs. Analysis of Tbr2+ TACs showed severe reduction of these cells in the dorsal V-SVZ of mutant mice in both density (Fig. 4G–I; 0.1629±0.01479 cells per 100 µm^2^ for *n*=3 controls and 0.0128±0.003706 cells per 100 µm^2^ for *n*=3 mutants; *P*-value=0.0006) and total number (Fig. S2N; 76.67±5.069 cells/SVZ for *n*=3 controls and 47.78±6.859 cells/SVZ for *n*=3 mutants; *P*-value=0.0276) in the dorsal V-SVZ of P7 *hGFAP-Cre;Sufu^fl/fl^* mice compared to controls. To examine if the reduction in Tbr2+ cells was due to the inability to proliferate, we double-labeled Tbr2+ cells with the proliferation marker Ki67(Fig. 4J,K). As with controls, Tbr2+ cells in the mutant dorsal V-SVZ co-labeled with Ki67 (Fig. 4L). Our quantification verified these observations in which no significant difference in the number of proliferating Tbr2+ cells between mutants and controls was evident (Fig. 4L; 42.8±3.811% of Tbr2+ cells in controls and 35.95±3.483% of Tbr2+ cells in mutants, *P*-value=0.2554; *n*=3 per genotype).

Taken together, our observations showed that loss of Sufu did not disrupt the ability of TACs to proliferate. However, the decrease in density of Gsx2+ and Tbr2+ TACs point to deficiencies in Type B1 cells to generate these precursors. It also appears that Tbr2+ TACs were severely affected. Given that Gsx2+ and Tbr2+ are derived from NSCs that originate from ventral and dorsal forebrain regions, respectively (Kowalczyk et al., 2009; López-Juárez et al., 2013), these observations reveal that Sufu differentially affects molecularly distinct subpopulations of Type B1 cells as determined by their embryonic origins.

### Delayed maturation of ependymal cells in the dorsal V-SVZ of P7 *hGFAP-Cre;Sufu^fl/fl^* mice

Given the reduction in Gsx2+ and Tbr2+ TACs in the dorsal V-SVZ, we wondered if loss of Sufu also affected the formation of non-proliferative ependymal cells. Ependymal cells control the structural integrity of the V-SVZ. These cells are arrange in a single-cell layer along the ventricular lining to form a barrier between the ventricle lumen and the brain parenchyma (Lim and Alvarez-Buylla, 2016). Although the majority of ependymal cells are specified and generated from RGCs at embryonic stages, their maturation into functional ependymal cells begins at birth and concludes by P20 (Spassky et al., 2005). Indeed at P7, we observed sparsely distributed ependymal cells, labeled with the ependymal cell marker S100β (S100β+), along the ventricular wall of the control dorsal V-SVZ (Fig. 4O). In contrast, in the P7 *hGFAP-Cre;Sufu^fl/fl^* dorsal V-SVZ we observed fewer S100β+ cells along the ventricular wall (Fig. 4M,N) and was significantly reduced compared to controls (Fig. 4O; 0.1748±0.0044 cells per 100 µm^2^ for *n*=3 controls and 0.0943±0.02559 cells per 100 µm^2^ for *n*=3 mutants; *P*-value=0.0363). Nevertheless, by P28, we observed neatly lined S100β+ ependymal cells along the ventricular wall of the control and *hGFAP-Cre;Sufu^fl/fl^* dorsal V-SVZ (Fig. S3). These findings indicate that despite the delay in maturation, Sufu deletion does not impede the formation and maturation of ependymal cells along the ventricular lining of the dorsal V-SVZ.

### Loss of Sufu downregulated Gli3 expression and Shh signaling activity

**Fig. 5.**
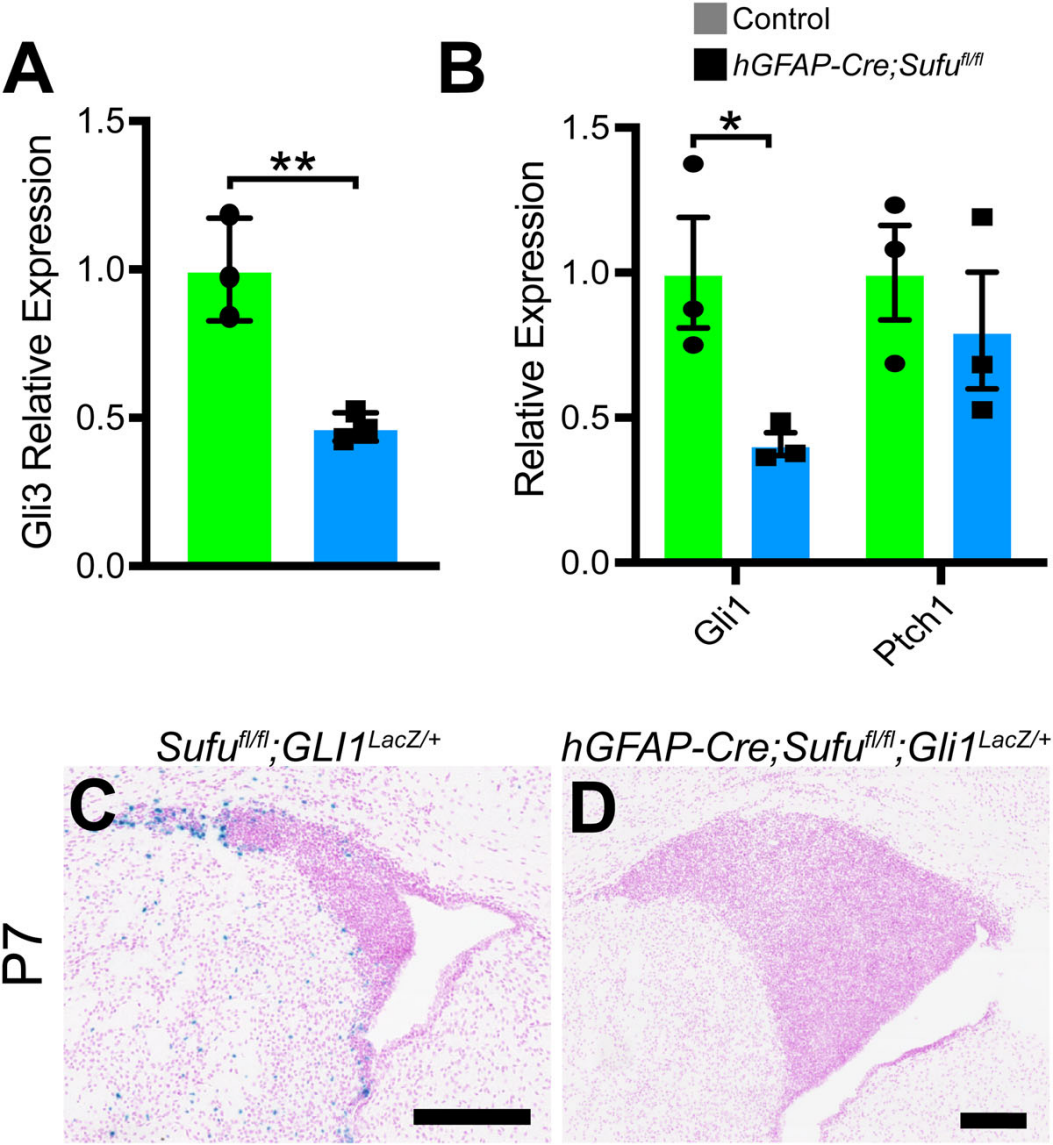
**Loss of Sufu results in downregulated Gli3 expression in the dorsal V-SVZ and did not ectopically activate Shh signaling.** (A) qPCR analysis of *Gli3* mRNA levels extracted from dissected V-SVZ shows that *Gli3* expression is significantly reduced in the P7 V-SVZ of *hGFAP-Cre;Sufu^fl/fl^* mice (*n*=3 controls/mutants). (B) qPCR analysis of Shh targets, *Gli1* and *Ptch1*, shows that *Gli1* expression is significantly reduced in the P7 V-SVZ of *hGFAP-Cre;Sufu^fl/fl^* mice compared to controls. However, levels of *Ptch1* expression were comparable between controls and mutant mice, indicating that Shh signaling activity is not increased in the dorsal V-SVZ of mice lacking Sufu (*n*=3 controls/mutants). (C,D) Shh-responsive cells, as detected by β-galactosidase activity (LacZ+) in mice carrying the Shh-reporter Gli1-LacZ, are largely absent in the dorsal V-SVZ of P7 *hGFAP-Cre;Sufu^fl/fl^* mice (D), whereas few LacZ+ cells were observed in the controls (C) (*n*=3 controls/mutants). Scale bars: 200 µm. *P-value ≤0.05; **P-value ≤0.03.

Similar to the P7 *hGFAP-Cre;Sufu^fl/fl^* V-SVZ, deletion of *Gli3* in NSCs of the developing brain cause expansion of the dorsal V-SVZ in neonatal mice (Petrova et al., 2013; Wang et al., 2014). We examined if changes in Gli3 levels occurred in the P7 *hGFAP-Cre;Sufu^fl/fl^* V-SVZ. Quantitative PCR analysis showed that *Gli3* mRNA was reduced in dissected V-SVZ of P7 *hGFAP-Cre;Sufu^fl/fl^* mice (Fig. 5A; 1±0.09995 relative expression level in controls, 0.4697±0.02742 levels in mutants, *n*=3 mice/genotype, *P*-value=0.0069). These findings suggest that loss of Sufu results in diminished expression of *Gli3* causing the defects observed in the *hGFAP-Cre;Sufu^fl/fl^* mice. As with mice lacking Gli3 in the V-SVZ (Wang et al., 2014), we did not observe ectopic activation of Shh signaling in the dorsal V-SVZ of the P7 *hGFAP-Cre;Sufu^fl/fl^* mice by visualizing cells that express LacZ under the control of the Gli1 promoter (LacZ+) (Ahn and Joyner, 2005). Surprisingly, far fewer LacZ+ cells were observed in the dorsal V-SVZ of P0 and P7 mutant mice (Fig. 5C,D and Fig. S4). This observation correlated with the reduced Gli1 expression in the dorsal V-SVZ of mutant mice (Fig. 5B; 1±0.1909 relative expression level in controls, 0.4089±0.03958 levels in mutants, *n*=3 mice/genotype, *P*-value=0.0387). Further supporting these observations, expression of the Shh target gene, Ptch1, was also comparable between control and mutant mice (Fig. 5B; 1±1628 relative expression level in controls, 0.8008±0.2008 levels in mutants, *n*=3 mice/genotype, *P*-value=0.4839). These results showed that Sufu deletion in the neonatal dorsal V-SVZ does not ectopically activate Shh signaling. Furthermore, these findings indicate that Shh signaling does not drive the uncontrolled expansion of Sox2+ Type B1 cells in the neonatal *hGFAP-Cre;Sufu^fl/fl^* dorsal V-SVZ.

### Normal migration and maturation of OB interneurons in the P28 *hGFAP-Cre;Sufu^fl/fl^* mice

**Fig. 6.**
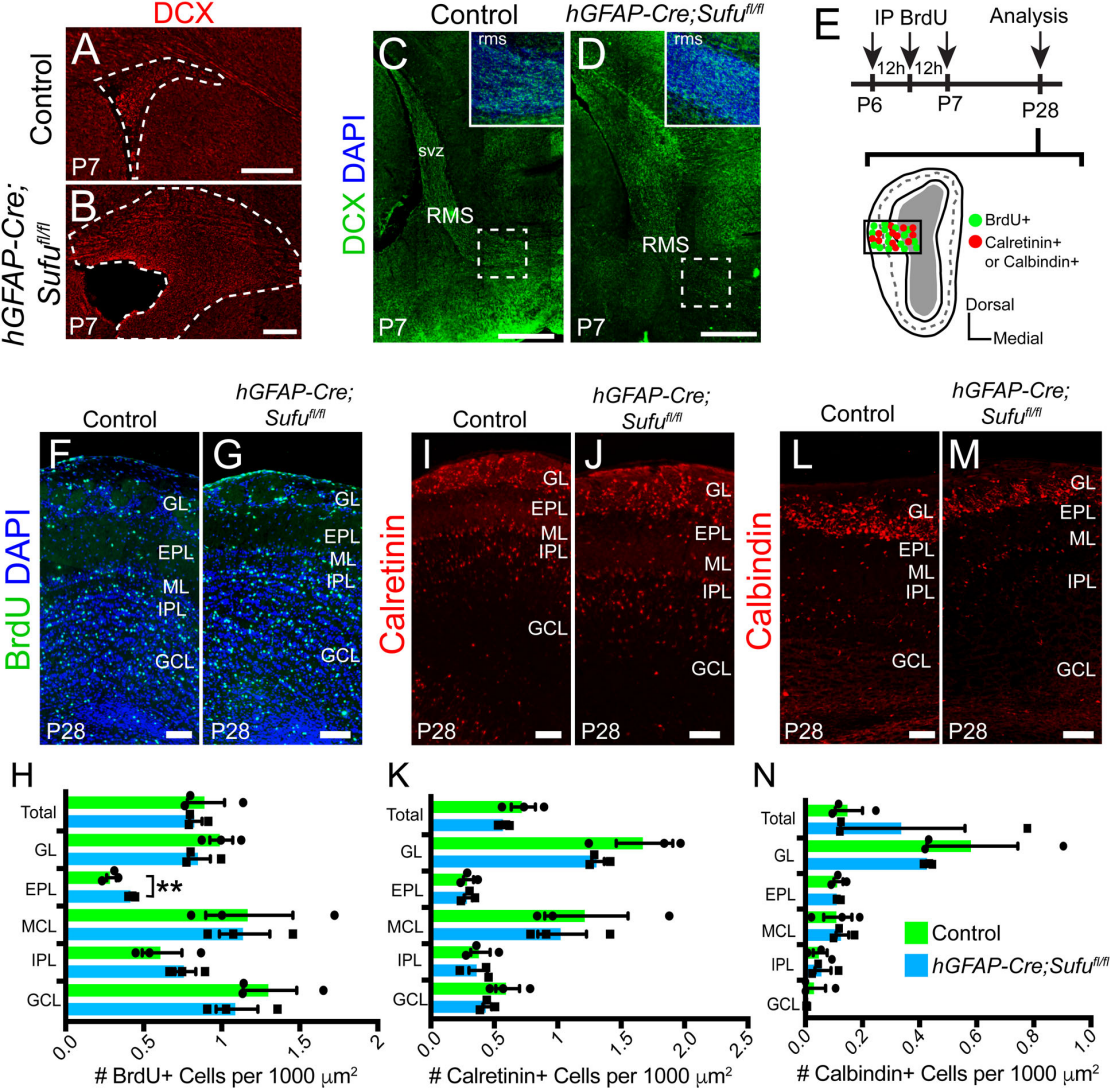
**Type A cells are produced and able to differentiate into interneuron subtypes in the P28 *hGFAP-Cre;Sufu^fl/fl^* OB.** (A–D) Immunofluorescence staining against DCX, which labels Type A cells, shows that Type-A cells are generated in the dorsal V-SVZ of the P7 control and mutant mice and are able to migrate through the RMS. Scale bars: (A,B) 250 µm, (C,D) 500 µm. (E) Schematic of the experimental design to identify the progeny of proliferating cells in the P7 dorsal V-SVZ. Three pulsed intraperitoneal injections at 12-h intervals of S-phase label. BrdU was administered starting at P6 to label proliferating cells in the V-SVZ and determine their localization and identity in the OB at P28. (F,G) Immunofluorescence staining against BrdU shows visible confirmation of proliferating Type A cells migrating into the OB layers of P28 *hGFAP-Cre;Sufu^fl/fl^* mice and controls. Scale bars: 100 µm. (H) Quantification of BrdU+ cells in each OB layer shows a significant increase in BrdU+ cells in external plexiform layer (EPL) of mutant mice compared to controls. However, no other significant increase was observed in the remaining layers of the OB (*n*=3 controls/mutants). (I,J) Immunofluorescence staining against the dorsal V-SVZ derived interneuron cell marker, Calretinin in the P28 OB. Scale bars: 100 µm. (K) Quantification of Calretinin+ cells in the OB confirms no significant difference in individual layers of the OB or as a whole (*n*=3 controls/mutants). (L,M) Immunofluorescence staining against ventral V-SVZ derived interneuron cell marker, Calbindin. Scale bars: 100 µm. (N) Quantification of Calbindin+ cells in the OB confirms there is no significant difference in individual layers of the OB or as a whole (*n*=3 controls/mutants). RMS, rostral migratory stream; OB, olfactory bulb; GL, glomerular layer; EPL, external plexiform layer; MCL, mitral cell layer; IPL, internal plexiform layer; GCL, granule cell layer; **P-value ≤0.03.

The dramatic increase in Type B1 cells and the decrease in TACs in the dorsal V-SVZ at P7, led us to investigate if the number of interneurons in the OB have been compromised. Immunostaining for Doublecortin (Dcx), a marker for immature neurons, showed Dcx-expressing (Dcx+) cells in the dorsal V-SVZ and the RMS indicating that TACs were able to differentiate into immature neurons (Fig. 6A–D). To confirm that proliferating TACs in the P7 dorsal V-SVZ differentiated into interneurons in the OB, we treated neonatal pups with three pulses of BrdU every 12 h beginning at P6 to efficiently label and fate map cells that were actively proliferating in the P7 V-SVZ cells (Fig. 6E). Results from these experiments showed that BrdU-labeled V-SVZ cells successfully migrated and integrated into various OB layers by P28 (Fig. 6F,G). We found that the total number of BrdU+ cells in the OB of control and mutant mice did not significantly differ (Fig. 6H, 0.08992±0.01195 cells per 100 µm^2^ for *n*=3 controls and 0.08334±0.004048 cells per 100 µm^2^ for *n*=3 mutants; *P*-value=0.6297), indicating that proper numbers of immature neurons originating from the P7 *hGFAP-Cre;Sufu^fl/fl^* V-SVZ migrated into the OB. Quantification of BrdU+ cells in each OB layer revealed a significant increase in BrdU+ cells in the external plexiform layer (EPL) of mutant mice compared to controls (Fig. 6H, 0.02938±0.003135 cells per 100 µm^2^ for *n*=3 controls and 0.04247±0.001264 cells per 100 µm^2^ for *n*=3 mutants; *P*-value=0.0180). However, no significant changes were observed in the number of BrdU+ cells of the granule cell layer (GCL) (0.1309±0.01715 cells per 100 µm^2^ for *n*=3 controls and 0.1098±0.01342 cells per 100 µm^2^ for *n*=3 mutants; *P*-value=0.3871), internal plexiform layer (IPL) (0.06176±0.01272 cells per 100 µm^2^ for *n*=3 controls and 0.07677±0.00666 cells per 100 µm^2^ for *n*=3 mutants; *P*-value=0.3550), or mitral cell layer (MCL) (0.1176±0.02794 cells per 100 µm^2^ for *n*=3 controls and 0.1147±0.01621 cells per 100 µm^2^ for *n*=3 mutants; *P*-value=0.9313), between controls and mutants. These findings indicate that despite the reduction in TACs in the P7 dorsal V-SVZ, Sufu deletion did not dramatically alter the number of interneurons in the OB at later stages.

To examine if specific interneuron populations were affected by defects in the dorsal V-SVZ of neonatal *hGFAP-Cre;Sufu^fl/fl^* mice, we immunostained for OB interneuron markers, Calretinin and Calbindin, both of which label the major principal OB neurons: the periglomerular cells and granule cells (Kosaka and Kosaka, 2005). As shown in Fig. 6I,J Calretinin-expressing (Calr+) cells were present across all OB layers of control and mutant P28 OB. Indeed, we did not find any difference in the total number of Calr+ cells in the OB as a whole (0.07293±0.009564 cells per 100 µm^2^ for *n*=3 controls and 0.05808±0.002665 cells per 100 µm^2^ for *n*=3 mutants; *P*-value=0.2090) or in each OB layer between controls and mutants (Fig. 6K). Additionally, we analyzed Calbindin-expressing (Calb+) interneurons, which are typically generated by neuronal precursors of the ventral V-SVZ (Merkle et al., 2007). We found no obvious difference in the distribution of Calb+ neurons across all OB layers (Fig. 6L,M). We also did not find any significant differences in the total number of Calb+ neurons between control and mutant OB (Fig. 6N, 0.3904±0.08594 cells per 100 µm^2^ for *n*=3 controls and 0.3273±0.08067 cells per 100 µm^2^ for *n*=3 mutants; *P*-value=0.5966), nor in any specific OB layer. Taken together, these findings showed that the dramatic expansion of the dorsal V-SVZ in the neonatal *hGFAP-Cre;Sufu^fl/fl^* mice did not inhibit the generation of properly specified and functional Type A cells. Indeed, as with control mice, these cells retained the capability to migrate and mature into specific interneuron subtypes in the OB of *hGFAP-Cre;Sufu^fl/fl^* mice.

### A greater number of proliferating Type B1 cells at P7 remained in the P28 dorsal V-SVZ of *hGFAP-Cre;Sufu^fl/fl^* mice

**Fig. 7.**
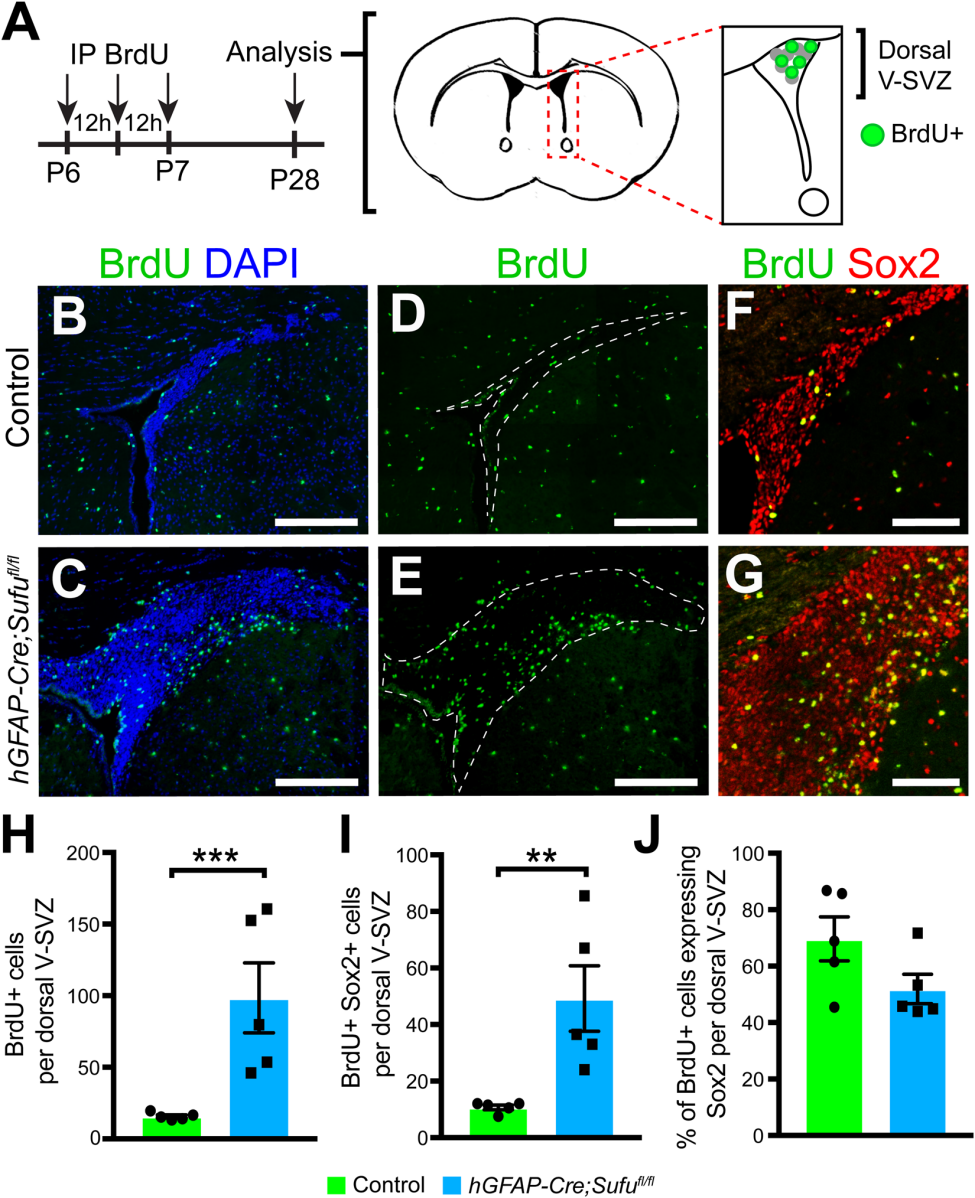
**Cells generated in the dorsal V-SVZ at P7 accumulate in the dorsal V-SVZ of P28 *hGFAP-Cre;Sufu^fl/fl^* mice.** (A) Schematic of the experimental design to identify the progenies of proliferating cells in the P7 dorsal V-SVZ. Three pulsed intraperitoneal injections at 12-h intervals of S-phase label. BrdU was administered starting at P6 to label proliferating cells in the V-SVZ and determine if any BrdU-labeled cells remained in the dorsal V-SVZ at P28. (B–E) Immunofluorescence staining against BrdU shows an obvious increase in the number of BrdU+ cells in the dorsal V-SVZ of the P28 *hGFAP-Cre;Sufu^fl/fl^* mouse. Scale bars: 200 µm. (F,G) Double-immunofluorescence staining shows that many BrdU+ cells co-labeled Sox2 in the P28 dorsal V-SVZ of control and mutant mice. Scale bars: 100 µm. (H) Quantification of BrdU+ cells in the V-SVZ demonstrated a significant decrease in *hGFAP-Cre;Sufu^fl/fl^* mice compared to controls (*n*=5 controls/mutants). (I,J) Quantification of BrdU+/Sox2+ cells in the V-SVZ indicates a significant increase in *hGFAP-Cre;Sufu^fl/fl^* mice compared to controls (I) although no difference is observed in the percentage of BrdU+/Sox2+ cells (J; *n*=5 controls/mutants). **P-value≤0.03; ***P-value≤0.01.

The lack of any significant changes in OB interneurons prompted us to further examine the fate of NSCs and TACs in the dorsal V-SVZ of *hGFAP-Cre;Sufu^fl/fl^* mice. We found that cells labeled with BrdU at P7 (Fig. 7A) remained in the dorsal V-SVZ of mutant mice at P28 unlike controls (Fig. 7B–E). Quantification showed a dramatic increase in BrdU+ cells in the P28 mutant dorsal V-SVZ compared to controls (Fig. 7H; 15.7±1.099 cells per dorsal V-SVZ for *n*=5 controls and 98.45±24.39 cells per dorsal V-SVZ for *n*=5 mutants; *P*-value=0.0095). We also found a significantly higher number of BrdU+ cells expressing Sox2 (Fig. 7F,G and I 10.7±0.8456 cells per dorsal V-SVZ for *n*=5 controls and 49.2±11.6 cells per dorsal V-SVZ for *n*=5 mutants; *P*-value=0.0107). Although we found a significantly higher number of BrdU+ cells co-expressing Sox2 in mutants, we did not find a disproportionate increase of double-labeled cells (Fig. 7J 69.66±7.761% of *n*=5 controls, 51.91±5.226% of *n*=5 mutants, *P*-value=0.0944). These findings indicate that many proliferating Type B1 cells in the P7 dorsal V-SVZ remained in this region at P28, likely contributing to the enlarged size of the P28 *hGFAP-Cre;Sufu^fl/fl^* dorsal V-SVZ.

### Increased cell death in the P7 *hGFAP-Cre;Sufu^fl/fl^* mice dorsal V-SVZ

**Fig. 8.**
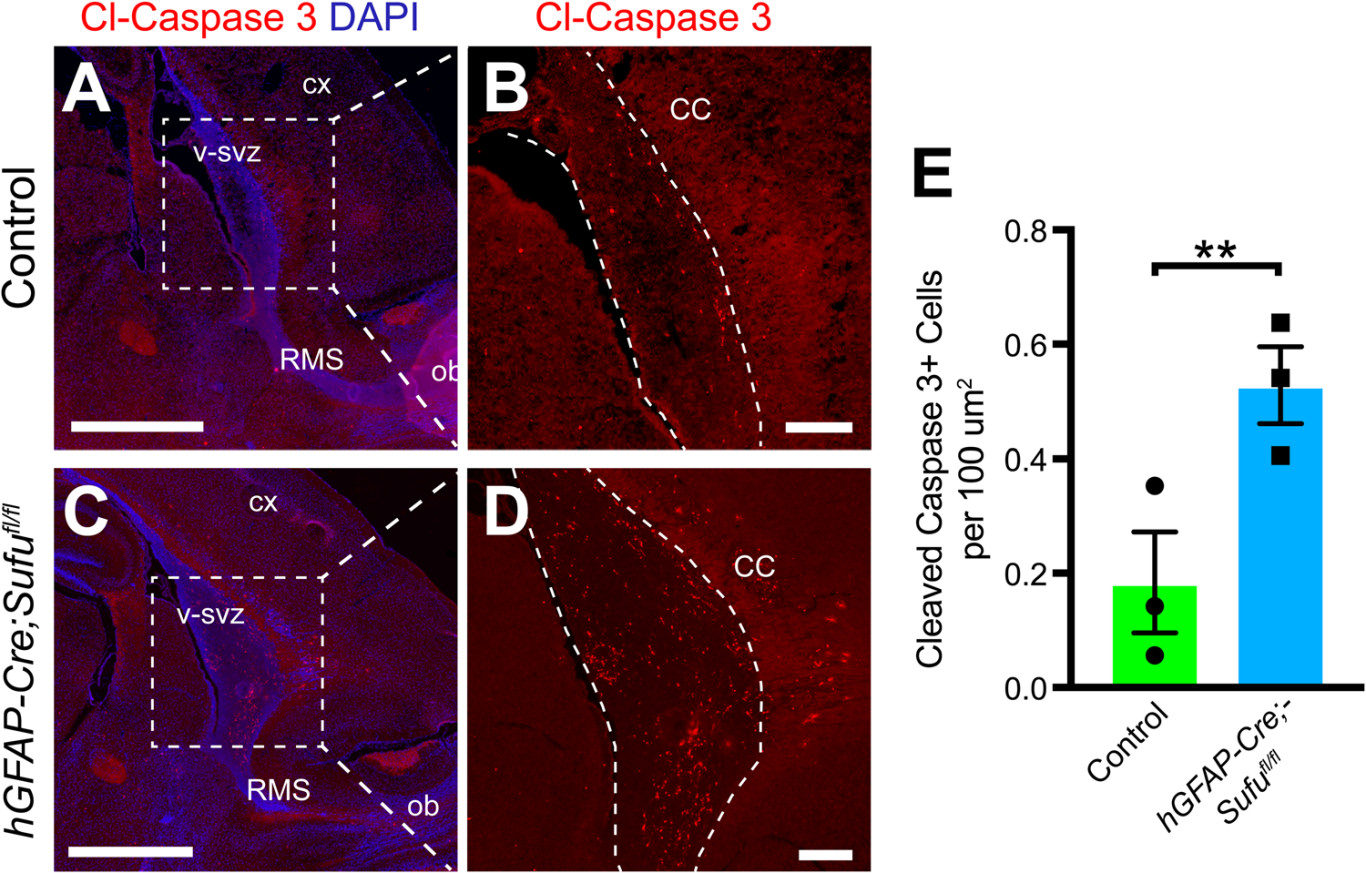
**Increase in cell death along the SVZ of the P7 *hGFAP-Cre;Sufu^fl/fl^* mice.** (A–D) Immunofluorescence staining against cell death marker, Cl-Casp3+, shows an observable difference in the number of Cl-Casp3+ in the dorsal V-SVZ of P7 *hGFAP-Cre;Sufu^fl/fl^* mice compared to controls. (E) Quantification of Cl-Casp3+ cells in the V-SVZ demonstrated a significant increase in P7 *hGFAP-Cre;Sufu^fl/fl^* mice compared to controls (*n*=3 controls/mutants). **P-value≤0.03. SVZ, subventricular zone; RMS, rostral migratory stream; OB, olfactory bulb; CX, cortex; CC, corpus callosum. Scale bars: (A,C) 1000 µm, (B,D) 200 µm.

We previously observed similar expansion of specific neocortical progenitors in the embryonic neocortex of conditional Sufu knockouts and found that many were unable to survive (Yabut et al., 2016). Therefore, we investigated if cells in the dorsal V-SVZ of the neonatal *hGFAP-Cre;Sufu^fl/fl^* mice were similarly unstable and became apoptotic. We conducted immunostaining against the cell death marker, cleaved Caspase-3 (Cl-Casp3), and observed many dying cells (Cl-Casp3+) along the dorsal V-SVZ of mutant mice whereas Cl-Casp3+ cells in the dorsal V-SVZ of control mice were less frequent (Fig. 8A–D). Indeed, quantification of Cl-Casp3+ cells reflected these observations and showed a significant increase in apoptotic cells in the dorsal V-SVZ of P7 *hGFAP-Cre;Sufu^fl/fl^* mice (Fig. 8E; 0.184±0.08815 cells per 100 µm^2^ for *n*=3 controls and 0.5282±0.0672 cells per 100 µm^2^ for *n*=3 mutants; *P*-value=0.0360). These findings suggest that despite the massive expansion of precursor cells in the dorsal V-SVZ, many of these cells failed to survive and differentiate into mature cell types.

## DISCUSSION

The postnatal V-SVZ is composed of multiple neuronal precursor populations that sustain lifelong neurogenesis in rodents. Our study provides insights into the molecular mechanisms involved in the formation of a molecularly distinct neurogenic domain, the dorsal V-SVZ. We showed that the cytoplasmic adaptor protein, Sufu, plays important roles in controlling precursor number and viability. Genetic ablation of Sufu in RG cells at late embryonic stages caused a dramatic expansion of the dorsal V-SVZ, but not the ventral V-SVZ. This expansion is due to the uncontrolled proliferation and organization of Sox2+ Type B1 cells, resulting in deregulated production of TACs via Gli3-dependent mechanisms, and independent of Shh signaling activity. Our novel findings establish a crucial role for Sufu in maintaining precursor populations in the neonatal dorsal V-SVZ.

We found that loss of Sufu at late embryonic stages did not disrupt the formation of the V-SVZ. Mice lacking Sufu formed anatomically distinct dorsal and ventral V-SVZ domains capable of generating predicted subpopulations of interneuron subtypes in the OB. This indicates that progenitor specification, as determined by their localization along the dorsoventral V-SVZ axis, was not severely disrupted. However, the dorsal V-SVZ was expanded in mice lacking Sufu, as a result of the persistent proliferation of Sox2+ Type B1 cells. These findings implied that loss of Sufu at late embryonic stages maintained NSCs in a highly proliferative state at neonatal stages, preventing their differentiation into Tbr2+ and Gsx2+ TACs. Thus, Sufu must play a role in modulating the cell cycle progression of NSCs in the dorsal V-SVZ to ensure a timely production of specific NSC lineages.

Loss of Sufu drastically reduced the production of Tbr2+ TACs. Tbr2+ TACs typically originate from RGCs in the embryonic neocortical progenitors whereas Gsx2+ neural progenitors that originate from the embryonic ganglionic eminence (Stenman et al., 2003; Brill et al., 2009) were significantly increased. We previously reported that loss of Sufu results in the increase in proliferation of neocortical progenitors (resulting in an increase in superficial layer projection neurons in the neocortex) and oligodendrogenesis in the E16.5 neocortex of hGFAP-Cre;Sufu-fl mice (Yabut et al., 2016; Winkler et al., 2018). Thus, two possibilities could explain the reduction in Tbr2+ TACs: 1) neocortical progenitors that generate V-SVZ NSCs were re-specified towards the gliogenic lineage, and/or 2) exhaustion of the neocortical RGC pool has occurred. This would prevent neocortical RGCs to generate Type B1 cells in the dorsal V-SVZ. Lineage tracing of embryonic neocortical progenitors will determine if proportions of RGCs in the E16.5 neocortex, a time at which Type B1 cells are thought to be specified (Fuentealba et al., 2015; Furutachi et al., 2015), failed to generate Type B1 cells in the neonatal V-SVZ. Nevertheless, these findings indicate that in addition to previously identified roles of Sufu in corticogenesis, Sufu also functions to ensure the proper production of dorsal V-SVZ NSCs generating specific TAC subtypes. Since Tbr2+ TACs uniquely generate glutamatergic OB neurons (Brill et al., 2009), further investigation is required to determine if glutamatergic OB neurons are significantly reduced or if physiological disturbances in the OB circuitry has occurred as a consequence of abnormal production TACs.

Sufu antagonizes Shh signaling by mediating the proteolytic processing of Gli transcription factors to either inhibit activator function or promote the formation of transcriptional repressor forms. In the developing forebrain, we have previously shown that Sufu acts to regulate the stability and processing of Gli2 and Gli3 proteins at early stages of corticogenesis that resulting in an increase in Gli2R and Gli3R levels, while it functions to promote the generation of Gli3R alone at later stages (Yabut et al., 2015). Here, we found that loss of Sufu affected Gli transcription in dorsal V-SVZ cells. We found that Gli1 and Gli3 mRNA levels were significantly reduced in the *hGFAP-Cre;Sufu^fl/fl^* mice V-SVZ. Gli3 is typically highly expressed in the dorsal V-SVZ to exert its repressor function during the establishment of the V-SVZ (Petrova et al., 2013; Wang et al., 2014). These findings showed that in the absence of Sufu, Gli3 transcription is not efficiently maintained in the neonatal V-SVZ. Loss of Sufu may have resulted in the repression of Gli3 expression by deregulation of yet unidentified transcription factors that typically promote Gli3 transcription, or indirectly caused by defects in Gli3 protein processing triggering other transcriptional repressors to inhibit Gli3 expression. Elucidating the molecular steps by which Sufu alters Gli3 expression and activity in dorsal V-SVZ NSCs could provide novel insights on the diverse mechanisms utilized by Sufu to control Gli3 function.

Deletion of Sufu and the subsequent reduction in Gli3 transcription in the neonatal *hGFAP-Cre;Sufu^fl/fl^* mice resulted in defects that phenocopy Gli3R-cKO mice (Wang et al., 2014). Similar to our findings, ectopic activation of Shh signaling did not occur in Gli3R-cKO V-SVZ at neonatal stages. This could be because responsiveness to Shh signals by neonatal dorsal V-SVZ cells, such as in NSCs and TACs, do not occur until after P7 (Ahn and Joyner, 2005; Palma et al., 2005; Wang et al., 2014). Supporting this, conditional deletion of Smo in V-SVZ NSCs at P0, using the mGFAP-Cre driver, does not cause any obvious proliferation defects in the V-SVZ until after P15 (Petrova et al., 2013). This would indicate that the Sufu-Gli3 regulatory axis alone is critical in the control of progenitor populations in the dorsal V-SVZ niche, independent of its canonical roles as regulators of Shh signaling activity. Indeed, previous studies have shown that Gli3R functions to regulate gp130/STAT3 signaling in NSCs at early neonatal stages for proper establishment of the V-SVZ niche (Wang et al., 2014).

In summary, our studies identified multiple roles for Sufu in establishing appropriate cell number and identity in the neonatal dorsal V-SVZ. We found that Sufu maintains neurogenic precursor populations in the dorsal V-SVZ via regulation of Gli3. These findings underscore the importance of Sufu as a key regulator of stem/progenitor populations not only in the developing embryonic forebrain but also in establishing postnatal neurogenic niches such as the V-SVZ. These results have potential implications in how neural stem/progenitor populations are established and sustained in the postnatal neurogenic niche, how defects in proliferation could predispose these cells to a number of neurological diseases and malignancies, and provide insights on potential molecular strategies that can be utilized for regenerative therapies.

## MATERIALS AND METHODS

### Animals

Mice carrying the floxed Sufu allele (Sufu^fl^) were kindly provided by Dr Chi-Chung Hui (University of Toronto) and were genotyped as described elsewhere (Pospisilik et al., 2010). The hGFAP-Cre (Stock #004600) was obtained from Jackson Laboratories (Bar Harbor, ME, USA). Mice designated as controls did not carry the *hGFAP^Cre^* transgene and may have either one of the following genotypes: *Sufu^fl/+^* or *Sufu^fl/fl^* (Fig. 1A). All mouse lines were maintained in mixed strains, and analysis included male and female pups from each age group, although sex differences were not included in data reporting. All animal protocols were in accordance to the National Institute of Health regulations and approved by the UCSF Institutional Animal Care and Use Committee (IACUC).

### Quantitative PCR

Total RNA was isolated from dissected V-SVZ of P7 mice using TRIzol™ Reagent (Thermo Fisher Scientific), according to the manufacturer's instructions, and each sample was reverse-transcribed using a SuperScript IV cDNA Synthesis Kit (Invitrogen). Quantitative PCR reactions were performed using a KAPA SYBR Fast qPCR Kit (KAPA Biosystems) with ROX as reference dye, and transcript expression was measured via Applied Biosystems 7500 Real-Time PCR System (Life Technologies). Expression levels of each gene were normalized to RNA polymerase II subunit A (polr2a) and calculated relative to the control. The following primers were used: Gli1 Fw: CCGACGGAGGTCTCTTTGTC; Gli1 Rv AACATGGCGTCTCAGGGAAG; Gli3 Fw: AAGCGGTCCAAGATCAAGC; Gli3 Rv: TTGTTCCTTCCGGCTGTTC; Ptch1 Fw:TGACAAAGCCGACTACATGC; Ptch1 Rv:AGCGTACTCGATGGGCTCT; Polr2a Fw: CATCAAGAGAGTGCAGTTCG; Polr2a Rv: CCATTAGTCCCCCAAGTTTG.

### Immunohistochemistry and BrdU-Labeling

Perfusion, dissection, immunofluorescence and Nissl staining were conducted according to standard protocols as previously described (Siegenthaler et al., 2009). Cryostat sections were air dried and rinsed 3× in PBS plus 0.2% Triton before blocking for 1 h in 10% normal lamb serum diluted in PBS with 0.2% Triton to prevent nonspecific binding. Primary antibodies were diluted in 10% serum diluted in PBS with 0.2% Triton containing 40,6-diamidino-2-phenylindole (DAPI); sections were incubated in primary antibody overnight at room temperature. The following antibodies were used: mouse anti-BrdU (1:50 dilution; BD Pharmingen, #347580; Franklin Lakes, NJ, USA), rabbit anti-Phospho-Histone H3 (1:250 dilution; Millipore, #06-570; Billerica, MA, USA), rabbit anti-Sox2 (1:1000 dilution; Abcam, #ab92494; Cambridge, UK), rabbit anti-Tbr2 (1:500 dilution; Abcam, #ab23345), rabbit anti-cleaved Caspase-3 (1:300 dilution; Cell Signaling Technology, #9661S; Madison, WI, USA), mouse anti-Calretinin (1:250 dilution; Millipore, MAB168), mouse anti-Ki67 (1:250 dilution; BD Biosciences, #550089; USA), rabbit anti-GSX2 (1:250 dilution; gift from Kenneth Campbell; Toresson et al., 2000; mouse anti-Olig2; 1:250 dilution; Millipore, #MABN50), rabbit anti-Calbindin (1:1000 dilution; Swant, #CB-38; Switzerland), rabbit anti-Pdgfra (1:1000; gift from William Stallcup; Nishiyama et al., 1996; rabbit anti-Doublecortin; 1:250 dilution; Abcam, #ab18723), mouse anti-S100β (1:100; Sigma-Aldrich, #S2532; St. Louis, MO, USA). For 5-bromo-2-deoxyuridine (BrdU, Sigma-Aldrich, #10280879001) labeling, early postnatal mice were treated with 50 µg/g BrdU by intraperitoneal injection at P0-P1 prior to dissection at P7-P8. For BrdU-labelling at P28, mice were treated with 50 µg/g BrdU pulse by intraperitoneal injection from P6 to P7 every 12 h for 36 h for a total of 3 BrdU treatments. To detect primary antibodies, we used species-specific Alexa Fluor-conjugated secondary antibodies (1:500; Invitrogen) in 1X PBS-T for 1 h at room temperature, washed with 1X PBS, and coverslipped with Fluoromount-G (Southern Biotech).

### Image analysis and acquisition

Images were acquired using a Nikon E600 microscope equipped with a QCapture Pro camera (QImaging) or Zeiss Axioscan Z.1 (Zeiss, Thornwood, NY, USA) using the Zen 2 blue edition software (Zeiss). NIH ImageJ was used to quantify raw, unedited images. All analyses were conducted in at least two to three 20-µm-thick sections that were histologically matched at the rostral-caudal level between genotypes.

V-SVZ analysis: for measurement of VZ/SVZ thickness, the length of densely populated cell or DAPI+ regions adjacent to the lateral ventricles was measured and designated as the dorsal V-SVZ or ventral V-SVZ as defined in Fig. 1B. DAPI-dense regions were also used to define and measure the SVZ, RMS, and the OB to quantify BrdU-localization along sagittal sections. S100β+ cells were counted by measuring a 25-µm-thick region from the ventricular lining of the dorsal V-SVZ. Cells labeled with cell-specific markers were quantified within the dorsal V-SVZ to measure the number of cells per 100 µm^2^ or per V-SVZ.

OB analysis: a slice of the OB containing all layers, as designated according to their anatomical features (as defined in Fig. 6B), was used for cell quantification. Cells that express cell-specific markers (Calr+, Calb+, or BrdU+) were counted in each layer.

### Statistics

All experiments were conducted in triplicate with a sample size of *n*=3−6 embryos/animals per genotype. Unpaired Student’s *t*-test was conducted using Prism 7 (GraphPad) for pairwise analysis of control and mutant genotypes. Values of *P*<0.05 were considered statistically significant. Graphs display the mean±standard error of the mean (s.e.m.).

## Supplementary Material

Supplementary information

## References

[BIO039248C1] Ahn S. and Joyner A. L. (2005). In vivo analysis of quiescent adult neural stem cells responding to Sonic hedgehog. *Nature* 437, 894-897. 10.1038/nature03994 16208373

[BIO039248C2] Brill M. S., Snapyan M., Wohlfrom H., Ninkovic J., Jawerka M., Mastick G. S., Ashery-Padan R., Saghatelyan A., Berninger B. and Götz M. (2008). A dlx2- and pax6-dependent transcriptional code for periglomerular neuron specification in the adult olfactory bulb. *J. Neurosci.* 28, 6439-6452. 10.1523/JNEUROSCI.0700-08.2008 18562615 PMC3844782

[BIO039248C3] Brill M. S., Ninkovic J., Winpenny E., Hodge R. D., Ozen I., Yang R., Lepier A., Gascón S., Erdelyi F., Szabo G. et al. (2009). Adult generation of glutamatergic olfactory bulb interneurons. *Nat. Neurosci.* 12, 1524-1533. 10.1038/nn.2416 19881504 PMC2787799

[BIO039248C4] Codega P., Silva-Vargas V., Paul A., Maldonado-Soto A. R., DeLeo A. M., Pastrana E. and Doetsch F. (2014). Prospective identification and purification of quiescent adult neural stem cells from their in vivo niche. *Neuron* 82, 545-559. 10.1016/j.neuron.2014.02.039 24811379 PMC4360885

[BIO039248C5] Cotsarelis G., Sun T.-T. and Lavker R. M. (1990). Label-retaining cells reside in the bulge area of pilosebaceous unit: implications for follicular stem cells, hair cycle, and skin carcinogenesis. *Cell* 61, 1329-1337. 10.1016/0092-8674(90)90696-C 2364430

[BIO039248C6] Ellis P., Fagan B. M., Magness S. T., Hutton S., Taranova O., Hayashi S., McMahon A., Rao M. and Pevny L. (2004). SOX2, a persistent marker for multipotential neural stem cells derived from embryonic stem cells, the embryo or the adult. *Dev. Neurosci.* 26, 148-165. 10.1159/000082134 15711057

[BIO039248C7] Fuentealba L. C., Rompani S. B., Parraguez J. I., Obernier K., Romero R., Cepko C. L. and Alvarez-Buylla A. (2015). Embryonic origin of postnatal neural stem cells. *Cell* 161, 1644-1655. 10.1016/j.cell.2015.05.041 26091041 PMC4475276

[BIO039248C8] Furutachi S., Miya H., Watanabe T., Kawai H., Yamasaki N., Harada Y., Imayoshi I., Nelson M., Nakayama K. I., Hirabayashi Y. et al. (2015). Slowly dividing neural progenitors are an embryonic origin of adult neural stem cells. *Nat. Neurosci.* 18, 657-665. 10.1038/nn.3989 25821910

[BIO039248C9] Kosaka T. and Kosaka K. (2005). Structural organization of the glomerulus in the main olfactory bulb. *Chem. Senses* 30 Suppl. 1, i107-i108. 10.1093/chemse/bjh137 15738062

[BIO039248C10] Kowalczyk T., Pontious A., Englund C., Daza R. A. M., Bedogni F., Hodge R., Attardo A., Bell C., Huttner W. B. and Hevner R. F. (2009). Intermediate neuronal progenitors (basal progenitors) produce pyramidal-projection neurons for all layers of cerebral cortex. *Cereb. Cortex* 19, 2439-2450. 10.1093/cercor/bhn260 19168665 PMC2742596

[BIO039248C11] Lim D. A. and Alvarez-Buylla A. (2016). The adult ventricular-subventricular zone (V-SVZ) and olfactory bulb (OB) neurogenesis. *Cold Spring Harbor Perspect. Biol.* 8, a018820 10.1101/cshperspect.a018820 PMC4852803 27048191

[BIO039248C12] López-Juárez A., Howard J., Ullom K., Howard L., Grande A., Pardo A., Waclaw R., Sun Y.-Y., Yang D., Kuan C.-Y. et al. (2013). Gsx2 controls region-specific activation of neural stem cells and injury-induced neurogenesis in the adult subventricular zone. *Genes Dev.* 27, 1272-1287. 10.1101/gad.217539.113 23723414 PMC3690400

[BIO039248C13] Merkle F. T., Mirzadeh Z. and Alvarez-Buylla A. (2007). Mosaic organization of neural stem cells in the adult brain. *Science* 317, 381-384. 10.1126/science.1144914 17615304

[BIO039248C14] Nishiyama A., Lin X.-H., Giese N., Heldin C. H. and Stallcup W. B. (1996). Co-localization of NG2 proteoglycan and PDGF alpha-receptor on O2A progenitor cells in the developing rat brain. *J. Neurosci. Res.* 43, 299-314. 10.1002/(SICI)1097-4547(19960201)43:3<299::AID-JNR5>3.0.CO;2-E 8714519

[BIO039248C15] Palma V., Lim D. A., Dahmane N., Sánchez P., Brionne T. C., Herzberg C. D., Gitton Y., Carleton A., Alvarez-Buylla A. and Ruiz i Altaba A. (2005). Sonic hedgehog controls stem cell behavior in the postnatal and adult brain. *Development* 132, 335-344. 10.1242/dev.01567 15604099 PMC1431583

[BIO039248C16] Petrova R., Garcia A. D. R. and Joyner A. L. (2013). Titration of GLI3 repressor activity by Sonic hedgehog signaling is critical for maintaining multiple adult neural stem cell and astrocyte functions. *J. Neurosci.* 33, 17490-17505. 10.1523/JNEUROSCI.2042-13.2013 24174682 PMC3812512

[BIO039248C30] Pospisilik J. A., Schramek D., Schnidar H., Cronin S. J., Nehme N. T., Zhang X., Knauf C., Cani P. D., Aumayr K., Todoric J. et al. (2010). Drosophila genome-wide obesity screen reveals hedgehog as a determinant of brown versus white adipose cell fate. *Cell.* 140, 148-160. 10.1016/j.cell.2009.12.027 20074523

[BIO039248C17] Ramsbottom S. A. and Pownall M. E. (2016). Regulation of hedgehog signalling inside and outside the cell. *J. Dev. Biol.* 4, 23 10.3390/jdb4030023 27547735 PMC4990124

[BIO039248C18] Sakamoto M., Imayoshi I., Ohtsuka T., Yamaguchi M., Mori K. and Kageyama R. (2011). Continuous neurogenesis in the adult forebrain is required for innate olfactory responses. *Proc. Natl Acad. Sci. USA* 108, 8479-8484. 10.1073/pnas.1018782108 21536899 PMC3100923

[BIO039248C31] Siegenthaler J. A., Ashique A. M., Zarbalis K., Patterson K. P., Hecht J. H., Kane M. A., Folias A. E., Choe Y., May S. R., Kume T. et al. (2009). Retinoic acid from the meninges regulates cortical neuron generation. *Cell.* 139, 597-609. 10.1016/j.cell.2009.10.004 19879845 PMC2772834

[BIO039248C19] Spassky N., Merkle F. T., Flames N., Tramontin A. D., García-Verdugo J. M. and Alvarez-Buylla A. (2005). Adult ependymal cells are postmitotic and are derived from radial glial cells during embryogenesis. *J. Neurosci.* 25, 10-18. 10.1523/JNEUROSCI.1108-04.2005 15634762 PMC6725217

[BIO039248C20] Stenman J., Toresson H. and Campbell K. (2003). Identification of two distinct progenitor populations in the lateral ganglionic eminence: implications for striatal and olfactory bulb neurogenesis. *J. Neurosci.* 23, 167-174. 10.1523/JNEUROSCI.23-01-00167.2003 12514213 PMC6742158

[BIO039248C21] Toresson H., Potter S. S. and Campbell K. (2000). Genetic control of dorsal-ventral identity in the telencephalon: opposing roles for Pax6 and Gsh2. *Development* 127, 4361-4371. 11003836 10.1242/dev.127.20.4361

[BIO039248C22] Wang H., Kane A. W., Lee C. and Ahn S. (2014). Gli3 repressor controls cell fates and cell adhesion for proper establishment of neurogenic niche. *Cell Rep.* 8, 1093-1104. 10.1016/j.celrep.2014.07.006 25127137 PMC4151506

[BIO039248C23] Winkler C. C., Yabut O. R., Fregoso S. P., Gomez H. G., Dwyer B. E., Pleasure S. J. and Franco S. J. (2018). The dorsal wave of neocortical oligodendrogenesis begins embryonically and requires multiple sources of sonic hedgehog. *J. Neurosci.* 38, 5237-5250. 10.1523/JNEUROSCI.3392-17.2018 29739868 PMC5990977

[BIO039248C24] Yabut O. R. and Pleasure S. J. (2018). Sonic hedgehog signaling rises to the surface: emerging roles in neocortical development. *Brain Plast.* 3, 119-128. 10.3233/BPL-180064 30151337 PMC6091060

[BIO039248C25] Yabut O. R., Fernandez G., Huynh T., Yoon K. and Pleasure S. J. S. J. (2015). Suppressor of fused is critical for maintenance of neuronal progenitor identity during Corticogenesis. *Cell Rep.* 12, 2021-2034. 10.1016/j.celrep.2015.08.031 26387942 PMC4591209

[BIO039248C26] Yabut O., Ng H., Fernandez G., Yoon K., Kuhn J. and Pleasure S. (2016). Loss of suppressor of fused in mid-corticogenesis leads to the expansion of intermediate progenitors. *J. Dev. Biol.* 4, 29 10.3390/jdb4040029 28781964 PMC5542684

[BIO039248C27] Young K. M., Fogarty M., Kessaris N. and Richardson W. D. (2007). Subventricular zone stem cells are heterogeneous with respect to their embryonic origins and neurogenic fates in the adult olfactory bulb. *J. Neurosci.* 27, 8286-8296. 10.1523/JNEUROSCI.0476-07.2007 17670975 PMC6331046

[BIO039248C28] Zhuo L., Theis M., Alvarez-Maya I., Brenner M., Willecke K. and Messing A. (2001). hGFAP-cre transgenic mice for manipulation of glial and neuronal function in vivo. *Genesis* 31, 85-94. 10.1002/gene.10008 11668683

