# Supplementary material for: Suppressor of Fused regulates the proliferation of postnatal neural stem and precursor cells via a Gli3-dependent mechanism
Source: Biol Open. 2019 May 29;8(6):bio039248. doi: 10.1242/bio.039248 (PMC6602331; doi:10.1242/bio.039248)
Supplement: Supplementary information [file biolopen-8-039248-s1.pdf]

Biology Open (2019): doi:10.1242/bio.039248: Supplementary information

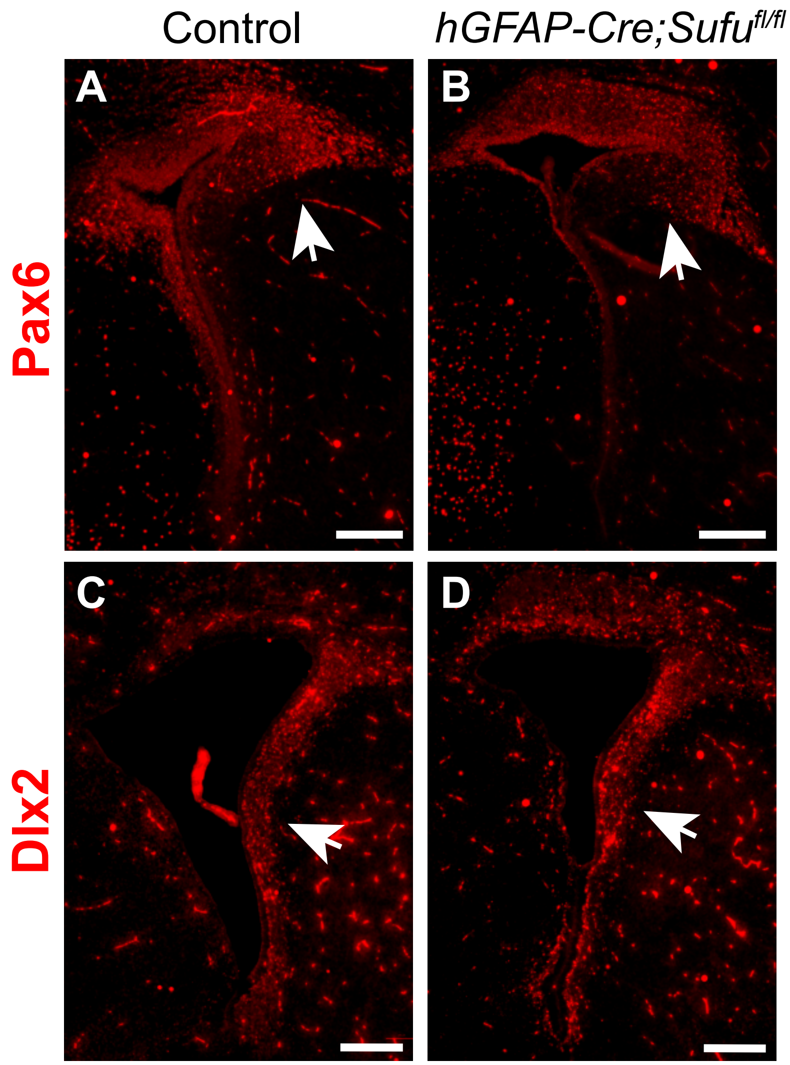

Figure S1. Establishment of the ventral and dorsal V-SVZ of the P0 *hGFAP-cre;Sufu$^{fl/fl}$* mouse.

(A, B) Immunofluorescence staining against dorsal forebrain progenitor marker, Pax6+, show restricted expression in the dorsal V-SVZ (arrows) and show no observable difference in expression ibetween mutants (B) and controls (A).

(C, D) Immunofluorescence staining against ventral V-SVZ marker, Dlx2, shows expression in ventral V-SVZ as expected an a partial increase in expression in the dorsal V-SVZ of the P0 ***hGFAP-cre;Sufu$^{fl/fl}$*** mouse compared to controls. Arrows show demarcation of expression boundaries. Scale bars represent 200 μm.

Biology Open • Supplementary information

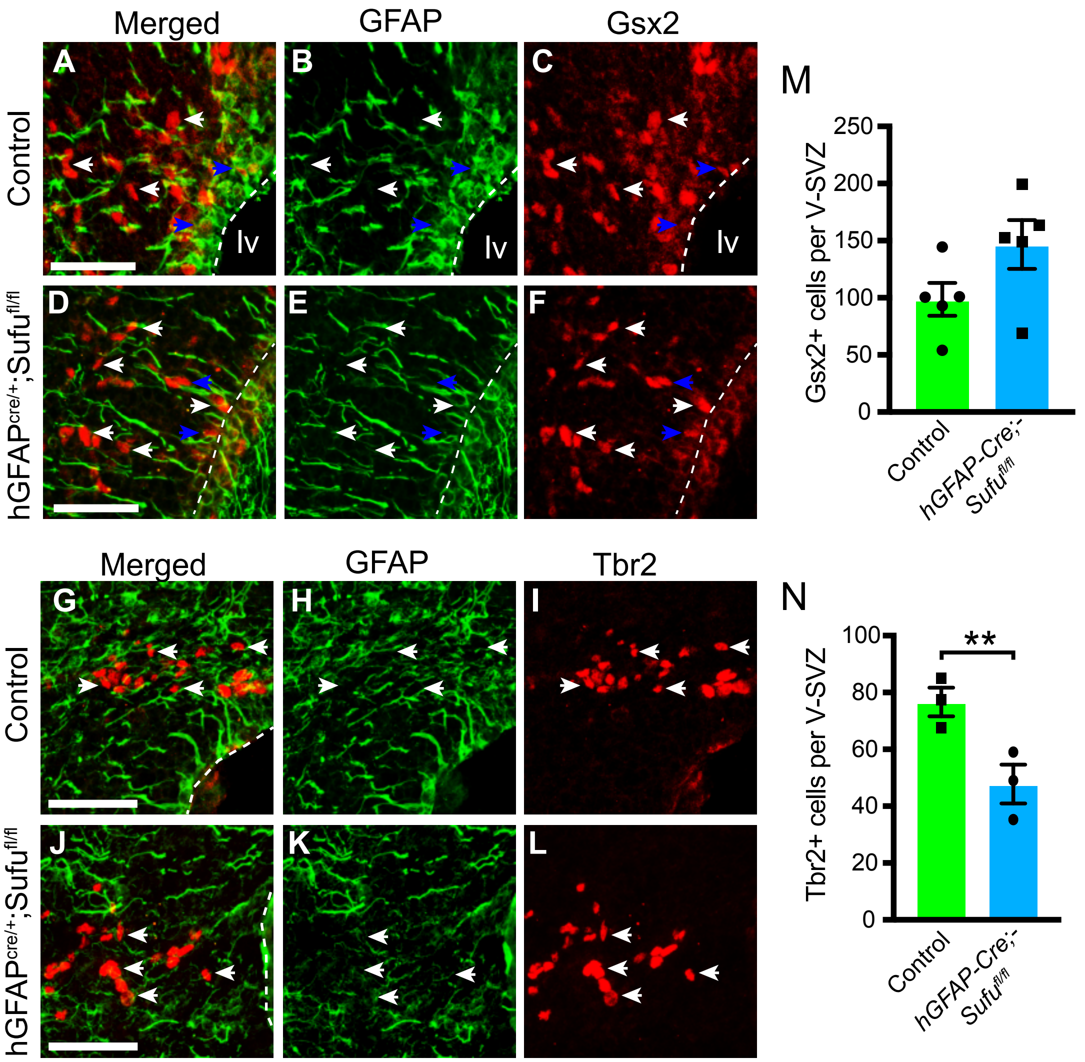

Figure S2. Gsx2 and Tbr2 -expressing cells do not express GFAP in the P7 dorsal V-SVZ of control and *hGFAP-cre;Sufu$^{fl/fl}$* mice.

(A-F) Immunofluorescence staining against GFAP and Gsx2 in the V-SVZ of P7 controls and *hGFAP-cre;Sufu$^{fl/fl}$* mice. Blue arrows indicate representative Gsx2+ cells expressing GFAP, many of which are located near the lateral ventricle (lv). White arrows indicate representative Gsx2+ cells that do not express GFAP, many of which are located away from the lateral ventricle. Scale bars represent 50 μm.

(G-L) Immunofluorescence staining against GFAP and Tbr2 in the V-SVZ of P7 controls and *hGFAP-cre;Sufu$^{fl/fl}$* mice. White arrows show that Tbr2+ cells are largely GFAP-negative. Scale bars represent 50 μm.

(M) Quantification of Gsx2+ cells in the V-SVZ demonstrated no difference between controls and *hGFAP-cre;Sufu$^{fl/fl}$* mice (n=5 controls/mutants) .

(N) Quantification of Tbr2+ cells in the V-SVZ indicates a significant decrease in *hGFAP-cre;Sufu$^{fl/fl}$* mice compared to controls (n=3 controls/mutants).

Biology Open • Supplementary information

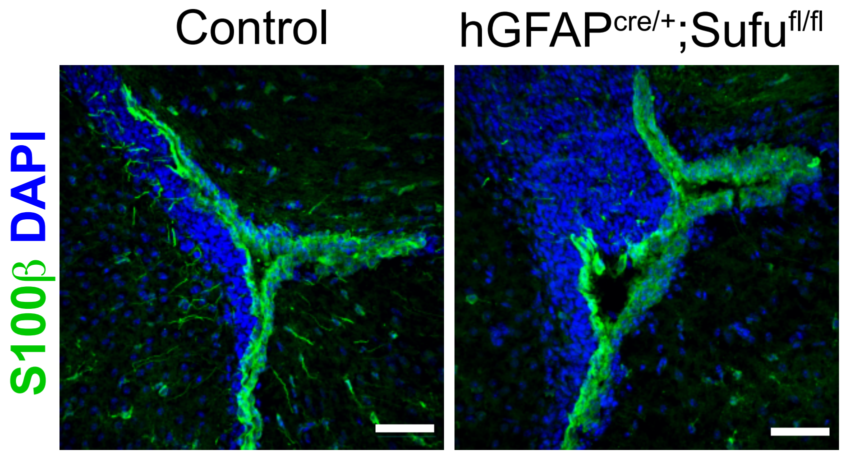

Figure S3. Ependymal cells are present in the P28 dorsal V-SVZ of control and *hGFAP-cre;Sufu$^{fl/fl}$* mice.
Immunofluorescence staining against the ependymal cell marker, S100β, show that the ependymal cells are present and uniformly distributed along the ventricular lining of the P28 control and mutant dorsal V-SVZ. Scale bars represent 50 μm.

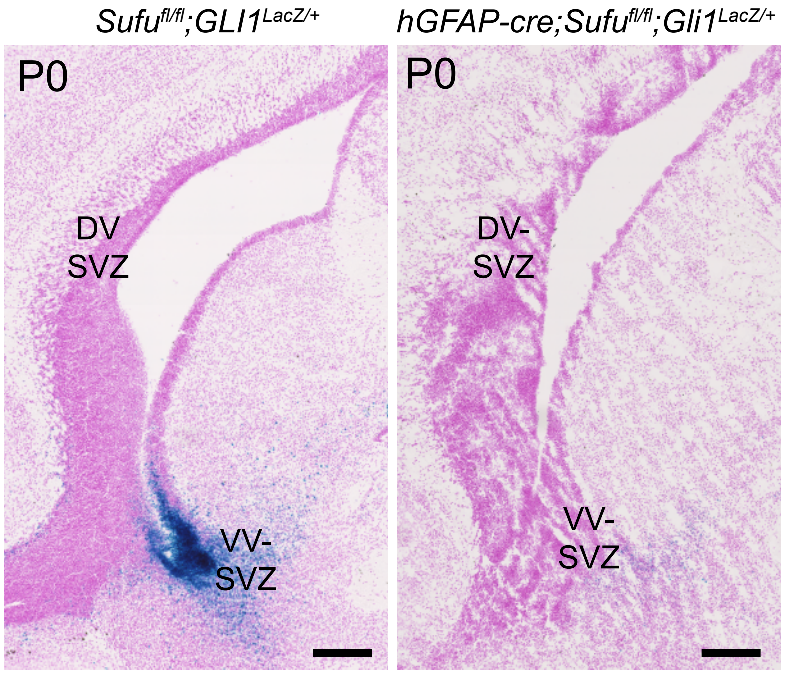

Figure S4. Reduced Gli1-LacZ activity in the P0 *Sufu$^{fl/fl}$;Gli1$^{LacZ/+}$* and *hGFAP-cre;Sufu$^{fl/fl}$;Gli1$^{LacZ/+}$* V-SVZ

LacZ staining of P0 *Sufu$^{fl/fl}$;Gli1$^{LacZ/+}$* and *hGFAP-cre;Sufu$^{fl/fl}$;Gli1$^{LacZ/+}$* mice shows remarkable reduction of Shh-responding cells in the ventral V-SVZ (VV-SVZ) of *hGFAP-cre;Sufu$^{fl/fl}$;Gli1$^{LacZ/+}$* mice. Scale bars represent 500 μm.

Biology Open • Supplementary information
